# Puffball‐Inspired Microrobotic Systems with Robust Payload, Strong Protection, and Targeted Locomotion for On‐Demand Drug Delivery

**DOI:** 10.1002/adma.202204791

**Published:** 2022-09-27

**Authors:** Xin Song, Rujie Sun, Richard Wang, Kun Zhou, Ruoxiao Xie, Junliang Lin, Dimitar Georgiev, Andrei‐Alexandru Paraschiv, Ruibo Zhao, Molly M. Stevens

**Affiliations:** ^1^ Department of Materials Department of Bioengineering Institute of Biomedical Engineering Imperial College London London SW7 2AZ UK; ^2^ Department of Computing UKRI Centre for Doctoral Training in AI for Healthcare Imperial College London London SW7 2AZ UK; ^3^ Department of Chemistry Imperial College London London SW7 2AZ UK; ^4^ Institute of Smart Biomaterials School of Materials Science and Engineering Zhejiang Sci‐Tech University Hangzhou Zhejiang 310018 China

**Keywords:** bioinspired systems, controlled release, intelligent microrobots, magnetic actuation, targeted therapy

## Abstract

Microrobots are recognized as transformative solutions for drug delivery systems (DDSs) because they can navigate through the body to specific locations and enable targeted drug release. However, their realization is substantially limited by insufficient payload capacity, unavoidable drug leakage/deactivation, and strict modification/stability criteria for drugs. Natural puffballs possess fascinating features that are highly desirable for DDSs, including a large fruitbody for storing spores, a flexible protective cap, and environmentally triggered release mechanisms. This report presents a puffball‐inspired microrobotic system which incorporates an internal chamber for loading large drug quantities and spatial drug separation, and a near‐infrared‐responsive top‐sealing layer offering strong drug protection and on‐demand release. These puffball‐inspired microrobots (PIMs) display tunable loading capacities up to high concentrations and enhanced drug protection with minimal drug leakage. Upon near‐infrared laser irradiation, on‐demand drug delivery with rapid release efficiency is achieved. The PIMs also demonstrate translational motion velocities, switchable motion modes, and precise locomotion under a rotating magnetic field. This work provides strong proof‐of‐concept for a DDS that combines the superior locomotion capability of microrobots with the unique characteristics of puffballs, thereby illustrating a versatile avenue for development of a new generation of microrobots for targeted drug delivery.

## Graphical abstract

Inspired by features of natural puffball fungi, including a fruitbody to store trillions of spores and a barrier cap to provide payload protection and stimuli‐triggered release, a bioinspired microrobotic system with an internal chamber and near‐infrared‐responsive sealing layer is presented for drug delivery. The microrobots show robust payload capacity, strong payload protection, on‐demand release, and targeted locomotion.

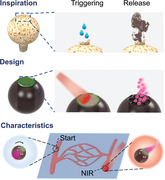

## 1 Introduction

Drug delivery systems (DDSs) are widely used for optimizing the concentration and release profile of drugs at specific locations while minimizing toxicity and side effects. Various targeted DDSs like liposomes,^[^ ^1^ ^]^ macromolecular conjugates,^[^ ^2^ ^]^ extracellular microparticles,^[^ ^3^ ^]^ micelles,^[^ ^4^ ^]^ framework nucleic acids,^[^ ^5^ ^]^ and biodegradable nanoparticles^[^ ^6^ ^]^ improve therapeutic outcomes by targeting specific ligand–receptor binding or enhancing permeability and retention (EPR) effects.^[^ ^7^ ^]^ However, without active propulsion mechanisms, the specificity of these passive DDSs is limited as their accumulation and distribution are primarily dictated by natural fluid circulation and diffusion concentration gradients. Recently developed active DDSs, such as implantable systems,^[^ ^8^ ^]^ microneedles,^[^ ^9^ ^]^ microchips,^[^ ^10^ ^]^ and microrobots^[^ ^11^ ^]^ have been shown to overcome the shortcomings of conventional passive DDSs. Among these, next‐generation microrobots have drawn extensive attention for the promise of superior control over delivery and targeted release.^[^ ^11a^ ^]^ These programmable machines have robust scalability (from micrometers to millimeters) and can be noninvasively triggered to execute simultaneous orthogonal tasks, including precise navigation and locomotion through complex fluidic environments to access hard‐to‐reach tissues and heightened spatiotemporal resolution of active drug delivery.^[^ ^12^ ^]^

The ideal microrobotic DDS should meet four core requirements: 1) high loading capacity, 2) protection of drug from the external environment, 3) a controllable propulsion mechanism, and 4) on‐demand triggered release of cargo. Research over the last decade has advanced various aspects of microrobot design, including actuation mechanisms (such as chemical,^[^ ^13^ ^]^ magnetic,^[^ ^14^ ^]^ ultrasound,^[^ ^15^ ^]^ light,^[^ ^11b^ ^]^ electrostatic,^[^ ^16^ ^]^ and biohybrid^[^ ^17^ ^]^) and structures to enable controllable propulsion (such as microhelices,^[^ ^18^ ^]^ microrollers,^[^ ^19^ ^]^ Janus microparticles,^[^ ^13^ ^]^ microbowls,^[^ ^20^ ^]^ porous microrobots,^[^ ^14^ ^]^ and other lithography based or 3D printed shapes^[^ ^15^ ^]^). Despite this progress, the challenges of high loading capacity, cargo protection, and on‐demand cargo delivery remain largely unmet.

First, the drug loading capacity of microrobots is limited because typical engine modules (like Zn^[^ ^21^ ^]^ or Mg^[^ ^11a^ ^]^ propellant engine segments and magnetic microbodies^[^ ^19^ ^]^) occupy a substantial volume within the microrobot. In turn, this limited loading capacity restricts treatment efficacy despite improved targeting compared to passive DDSs.^[^ ^22^ ^]^ Due to the insufficient internal volume for drug loading, surface coating^[^ ^11^, ^20^, ^23^ ^]^ or grafting^[^ ^13^, ^18^, ^19^, ^24^ ^]^ is a popular alternative drug‐loading strategy for microrobots. However, these approaches often exhibit poor drug protection as, during the transport process, the surface‐bound drugs may interact with existing inhibitors or the complex fluid environment, resulting in premature in vivo inactivation. One strategy to alleviate this issue is mixing the therapeutic agents with the microrobot bodies, such as within the resins used for 3D printing^[^ ^25^ ^]^ or hydrogels.^[^ ^26^ ^]^ This, however, leads to other complications such as drug denaturation from exposure to lasers used during fabrication when mixed with 3D printing resins^[^ ^27^ ^]^ or drug leakage from the porous crosslinked network when incorporated into hydrogels. Indeed, the protection of the drug is a broader challenge affected by factors beyond drug loading strategies. Although environmental stimuli‐responsive microrobots have been developed to target areas like tumors,^[^ ^23b^ ^]^ stomach,^[^ ^28^ ^]^ and intestines,^[^ ^29^ ^]^ the trigger mechanism accuracy can be compromised by unpredictable changes in complex and unstable physiological environments. Although external stimuli (such as light) have been reported to trigger drug release from microrobots,^[^ ^11^, ^30^ ^]^ the drugs must undergo chemical modification to provide the stimuli‐responsiveness, during which process they require to maintain high stability and withstand the effects of these external stimuli.

**Figure 1:**
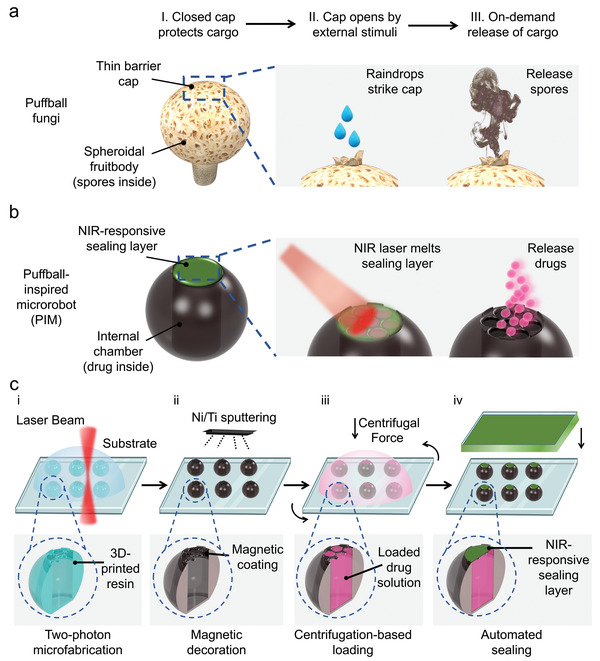
Design of the microrobotic system. a) Schematic illustration of puffball fungi. Numerous spores stored inside the spheroidal fruitbody are separated from the external environment by a thin barrier cap on the top. The barrier cap will open when stimulated by environmental factors such as raindrops, resulting in the immediate release of a spore cloud. b) Schematic diagram of PIMs. An internal chamber was designed to provide a large volume for drug loading, and a NIR‐responsive sealing layer was introduced to protect the drug while enabling on‐demand delivery. c) Schematic diagram for the fabrication process of the bioinspired microrobotic system: i) 3D microfabrication via two‐photon microprinting, ii) magnetic decoration via Ni/Ti sputtering, iii) centrifugation‐based loading, and iv) automated sealing. The cross‐section images of the microrobot illustrate individual features of a PIM generated in each step.

In nature, puffballs are an interesting fungus group with many unique characteristics analogous to payload‐carrying platforms (**Figure** **1**a). Unlike the open spore‐bearing gills of common fungi, puffball spores are produced and stored within spheroidal fruitbodies with enough internal capacity for trillions of spores.^[^ ^31^ ^]^ As such, the spores are physically protected from the surrounding environment by a thin and highly flexible barrier cap at the top of the spheroidal fruitbodies until they mature.^[^ ^32^ ^]^ External stimuli, such as raindrops or strong gusts of wind, will eventually impact the barrier cap to trigger the release of the mature spores in a puff cloud.^[^ ^33^ ^]^ These features are highly desirable for and relevant to the design of microrobotic DDS.

Herein, we report a bioinspired microrobotic system which mimics the features of puffball fungi and meets the core requirements for targeted drug delivery (Figure 1b). Inspired by the spheroidal fruitbody of puffballs, we designed an internal chamber within the microrobot to enable high drug payloads while offering protection from external environmental factors that may cause premature payload deactivation. Further, a near‐infrared (NIR)‐responsive top‐sealing layer was included to prevent payload leakage and premature payload inactivation during the delivery process, while enabling on‐demand release in the targeted area under external NIR triggering. The microrobot body was designed as a microroller with a magnetic coating to facilitate actuation and steering by external rotating magnetic fields. Once mobilized to the targeted area, a high‐penetration NIR laser was used as the stimulus to trigger the phase‐change of the top sealing layer, thereby resulting in on‐demand drug release. The puffball‐inspired microrobots (PIMs) presented here are shown to be an innovative drug delivery system with high payload capacity, effective payload protection, precise targeted navigation, and on‐demand controlled release. These collective features pave the way for future development into new and diverse drug delivery applications.

## 2 Results and Discussion

### 2.1 Design and Fabrication of the PIMs

Microrobotic DDSs with the desired properties were structurally and compositionally designed and fabricated in four steps: i) 3D microfabrication, ii) magnetic decoration, iii) centrifugation‐based loading, and iv) automated dip‐sealing (Figure 1c). We utilized two‐photon 3D microprinting to fabricate the structural features of the puffball‐inspired microrobot body (Figure 1c‐i). This is an emerging technique based on 3D laser lithography that has demonstrated high resolution and fidelity for creating complex microstructures.^[^ ^18^, ^34^ ^]^ Next, the microrobots were rendered magnetic and biocompatible through physical vapor deposition of nickel (Ni) and titanium (Ti) layers (Figure 1c‐ii), which is a modification strategy widely used to impart magnetic properties.^[^ ^24^, ^35^ ^]^ To load the drug into the microrobot, the drug solution was placed on top of the microrobots and forced into the internal space by physical centrifugation (Figure 1c‐iii). Finally, taking inspiration from the puffball barrier cap, we added a physical sealing layer on top of the microrobot to protect the drug payload from the external environment and potential premature deactivation (Figure 1c‐iv). To achieve this, the sealing material was first spin‐coated into a uniform film, which was then rapidly brought into contact with the top surface of the microrobot. Subsequent solidification of the sealing material, once below the melting temperature, resulted in a precisely formed thin sealing cap on the microrobot. This process was achieved automatically using a mechanical sensor. Importantly, the sealing layer was NIR‐responsive to facilitate noninvasive and on‐demand drug payload release for targeted delivery.

Microrobot structures must be capable of generating nonreciprocal motion to enable propulsion, especially at the micro scale where inertial forces are dominated by viscous forces in low Reynolds number environments.^[^ ^36^ ^]^ Toward this goal, spherical microrollers have recently gained popularity for microrobotic locomotion in cargo delivery applications.^[^ ^19^, ^35^, ^36^ ^]^ By exploiting surface frictional forces during rolling or surface‐walking motions, microrollers break the motion symmetry^[^ ^37^ ^]^ to achieve higher movement velocity with less impaired propulsion.^[^ ^19^ ^]^

**Figure 2:**
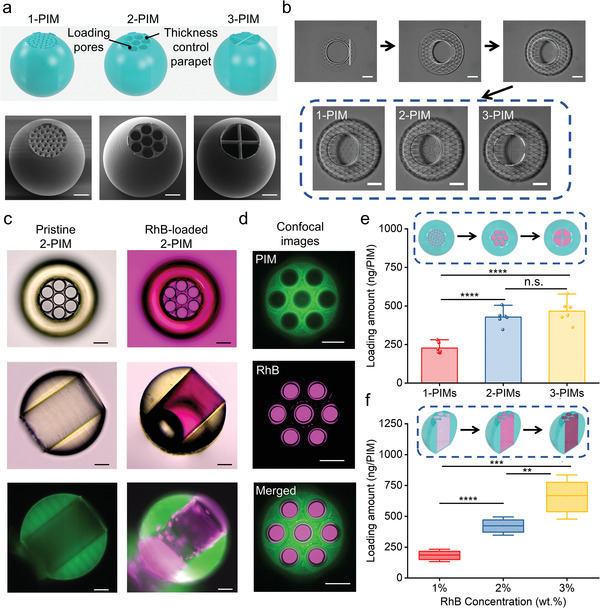
Fabrication and drug loading of the microrobotic system. a) Structure design of 1‐PIM, 2‐PIM, and 3‐PIM with different pore sizes (top). SEM images of single PIMs on substrates from the tilted view (bottom). From left to right, 1‐PIM, 2‐PIM, and 3‐PIM. b) Snapshots of the 3D microfabrication process of PIMs with different top porosity sizes and structures: construction of the PIM body (top) and three kinds of lids (bottom). c) Images of a pristine 2‐PIM (left) and a RhB‐loaded 2‐PIM (right). From top to bottom: top‐view bright‐field microscopy images (top), side‐view bright‐field microscopy images (middle), and side‐view fluorescence microscopy images (bottom). d) Confocal laser scanning microscopy images of a RhB‐loaded 2‐PIM. From top to bottom: photoresist channel (top), RhB channel (middle), and merged (bottom) images. e) RhB loading quantity for 1‐PIM, 2‐PIM, and 3‐PIM under the same loading process and loading solution (2 wt% RhB). All values are expressed as mean ± SD, *n* = 6, significance determined by an unpaired, two‐tailed t‐test, Student's t‐test, ^****^ *p* = 0.000015 for 1‐PIMs versus 2‐PIMs, ^****^ *p* = 0.000033 for 1‐PIMs versus 3‐PIMs, *p* = 0.319275 for 2‐PIMs versus 3‐PIMs. f) Loading quantity for 2‐PIMs using RhB solutions with different concentrations. All values are expressed as mean ± SD, *n* = 6, significance determined by an unpaired, two‐tailed *t*‐test, Student's *t*‐test, ^****^ *p* = 0.000002 for 1% versus 2%, ^**^ *p* = 0.003309 for 2% versus 3%, ^***^ *p* = 0.000106 for 1% versus 3%. Scale bars = 50 µm.

By combining the mechanical advantages of microroller designs with the bioinspired structures of puffballs, we envisaged a monolithic architecture for a PIM comprised of a spherical body for rolling propulsion with an internal chamber for drug loading (**Figure** **2**a). The cylindrical chamber was designed to provide a large space for drug loading and enhance the mechanical stability of the PIMs during the sealing process, while the top of the internal chamber was initially porous to allow drug loading via centrifugation before the NIR‐responsive sealing layer was applied. To investigate the effect of pore sizes on the drug loading and sealing processes, microrobots with small pores (diameter = 10 µm), medium pores (diameter = 40 µm), and large pores (diameters = 68 µm) (termed 1‐PIM, 2‐PIM, and 3‐PIM, respectively) were fabricated and compared. A specific parapet‐like feature was further fabricated around the pores to control the thickness of the NIR‐responsive sealing layer and the kinetics of the on‐demand phase change during payload release. The PIMs were subsequently 3D microfabricated using a two‐photon polymerization technique (Figure 2b). IP‐S photoresists, which were designed for Nanoscribe printing, were selected as the material for microrobot fabrication due to their proven handleability, high resolution, biocompatibility, and fidelity at the micro‐scale.^[^ ^34^ ^]^ The fabricated PIMs exhibited size uniformity, surface smoothness, and feature fidelity verified using scanning electron microscopy (SEM) (Figure 2a and Figures S1–S3, Supporting Information). In addition, the fabrication of various sizes of PIMs is highly scalable benefiting from the variety of options offered by the 3D microfabrication technique. To showcase this advantage, PIMs with a wide size range were fabricated and exhibited great structural integrity, indicating that the PIMs can serve as a versatile platform for a range of different potential clinical scenarios (Figure S4, Supporting Information).

### 2.2 Drug Loading of the PIMs

After confirming the successful fabrication of the PIMs, the drug loading capabilities were evaluated. Rhodamine B (RhB) was selected as the model drug cargo due to its fluorescent properties and wide use as a small‐molecule drug analog for microrobotic drug delivery applications.^[^ ^21^, ^38^ ^]^ Unlike existing drug loading strategies, we employed a centrifugation process to load drugs into the internal chamber of PIMs. Under bright‐field microscopy, the pristine PIM possessed clear visible pores on the top layer and a colorless empty internal chamber; the internal chamber was pink following centrifugal loading, indicating successful loading of the RhB solution (Figure 2c and Figure S5, Supporting Information). A small air bubble within the chamber was observed, which could be ascribed to the evaporation of the loaded cargo solution. The loading was confirmed under fluorescence and confocal microscopy. The photoresist exhibited green fluorescence signals, while the loaded RhB cargo in the PIM chamber emitted bright red fluorescence (Figure 2c,d). These results verified that the RhB drug cargo was successfully and selectively loaded into the protective internal chamber of PIMs through the pores, as opposed to absorption or coating onto the surface as seen in the microrobots of other studies.^[^ ^11^, ^20^, ^23^ ^]^

To confirm loading quantities within PIMs, specially designed microrobots without bottom layers were fabricated to allow loaded PIMs to fully release their cargo and facilitate loading quantification using a standard curve from ultraviolet–visible (UV–vis) spectroscopy (Figures S6 and S7, Supporting Information). Then, the relationship between centrifugation parameters and loading properties was studied. The primary purpose of employing the centrifugation loading method is to break surface tension and displace the air within the chamber with the drug solution. The PIMs will be fully loaded quickly once the surface tension is broken due to the high volume of drug solution placed on top, so increasing the centrifuge speed would not improve the loading amount significantly in this case (Figure S8a, Supporting Information). Therefore, a multicycle centrifugation loading strategy was applied to dramatically increase the loading amounts (Figure S8b, Supporting Information). A centrifugation loading process of 859 rcf and 7 cycles was chosen in this study. Furthermore, we evaluated different pore sizes and initial RhB concentrations to optimize the drug‐loaded quantity. Drug loading amounts showed an increasing trend with larger pore sizes on the PIMs, and 1‐PIMs showed significantly reduced loading compared to 2‐PIMs and 3‐PIMs (^****^ *p* = 0.000015 for 1‐PIMs vs 2‐PIMs, ^****^ *p* = 0.000033 for 1‐PIMs vs 3‐PIMs) (Figure 2e). This indicated that, under the same centrifugation process, the drug solutions passed through smaller pores less efficiently. Interestingly, there was no significant difference in loading amounts between 2‐PIMs and 3‐PIMs (*p* = 0.319275 for 2‐PIMs vs 3‐PIMs), indicating that increasing pore sizes had a diminishing effect on increasing drug loading quantities beyond a certain size. Using 2‐PIMs as a model, we further investigated the effect of initial RhB concentration on the final loading quantity (where loading amount refers to the weight of loaded RhB in the internal chamber of PIM). Since we only used 2‐PIMs with the same loaded volume of RhB, only the concentration of RhB dictated the loading amount. As expected, drug loading quantities increased significantly from 183.23 ± 33.39 ng per PIM to 656.17 ± 119.31 ng per PIM when the initial RhB concentration was increased from 1% to 3% (Figure 2f). This highlighted a significantly higher loading amount per single magnetic microrobot compared with previous literature in the same size scale.^[^ ^11^, ^24^ ^]^ Importantly, these results highlight the ability of PIMs to possess ultra‐high payload potential using high‐solubility drugs.

Additionally, compared with previous loading methods, the centrifugation loading strategy for PIMs was versatile for cargos with various formulations. For instance, a biocompatible polymer such as poly(ethylene glycol) (PEG) could be loaded into the PIM with a full payload in the internal chamber without any air bubbles (Figure S9a, Supporting Information). The results indicated that the PIMs were capable of simultaneously loading small‐molecule payloads as well as polymeric payloads without the need for complex modification methods, which also addressed the current challenge of loading payloads with different molecular weights. Moreover, other compounds such as dimethyl sulfoxide (DMSO) could also be loaded (Figure S9b, Supporting Information), which demonstrated the flexibility of adjusting the properties of loaded drugs by regulating the formula of the drug solution and the potential to load poorly soluble drugs using various solvents.

### 2.3 Drug Protection of the PIMs

Strong payload protection is one of the unmet challenges in drug delivery applications using microrobots. Inadequate designs result in premature drug deactivation and payload losses during transport. To address this, the microrobotic structure herein takes inspiration from the thin flexible cap on puffballs to incorporate a sealing layer covering the pores to facilitate drug protection. There are several requirements for optimizing the sealing layer materials. Firstly, the material should possess a suitable phase change property to allow controlled opening and drug release at the target site. In our design, an NIR laser was used to melt the sealing layer and trigger on‐demand drug release. Therefore, for biological applications, the sealing material must melt quickly in the NIR photothermal temperature range (around 50 °C). Next, the material should also be biocompatible to minimize immune reactions as melted material may separate from the microrobot body and remain circulating in the body. The material should also demonstrate strong adhesion with the top of the microrobot to prevent drug payload loss during locomotion, while maintaining a thin film coating to avoid interference with the locomotive mechanics of the microrobot. Finally, the sealant material must also be waterproof to prevent moisture ingress, which may reduce the concentration of the loaded drugs and/or cause drug leakage. After systematic evaluation of different types of sealing materials (Table S1, Supporting Information), polycaprolactone (PCL) diol with a low melting point (around 53 °C, Figure S10, Supporting Information) was found to be the most suitable material that met all the aforementioned requirements.

**Figure 3:**
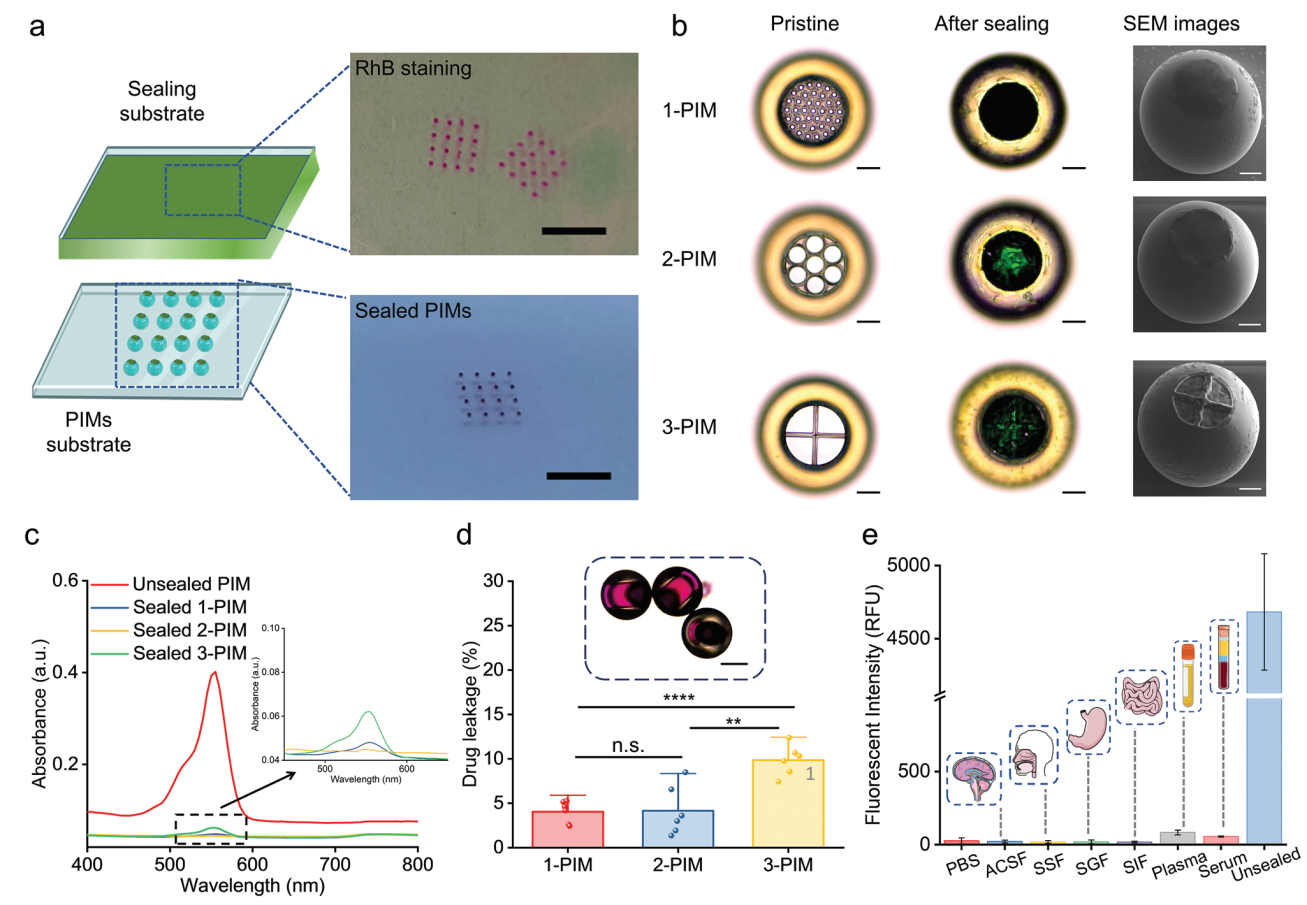
Drug protection ability of the microrobotic system. a) Schematic of the mechanically controlled sealing process (left). The sealing material was first spin‐coated into a uniform film and then rapidly brought into contact with the top surface of the microrobot. Subsequent solidification of the sealing material resulted in a precisely formed thin sealing cap on the microrobot. Images (right) show the physical transfer of RhB staining on the sealing substrate (top) and the sealed PIMs (bottom). Scale bars = 5 mm. b) Images of 1‐PIM, 2‐PIM, and 3‐PIM (top to bottom) before and after sealing. From left to right: bright‐field microscopy images of PIMs before sealing (left), bright‐field microscopy images of PIMs after sealing (middle), and SEM images of PIMs after sealing (right). Scale bars = 50 µm. c) UV–Vis spectra quantifying RhB leakage in incubation solution using PIMs with or without sealing layers. The inset figure shows the UV–Vis spectra of sealed PIMs. d) Drug leakage of 1‐PIM, 2‐PIM, and 3‐PIM designs. All values are expressed as mean ± SD, *n* = 6, significance determined by an unpaired, two‐tailed *t*‐test, Student's *t*‐test, *p* = 0.929146 for 1‐PIMs versus 2‐PIMs, ^****^ *p* = 0.000051 for 1‐PIMs versus 3‐PIMs, ^**^ *p* = 0.001655 for 2‐PIMs versus 3‐PIMs. The inset optical image shows no RhB leakage from sealed 2‐PIMs. Scale bar = 100 µm. e) Fluorescence intensity of RhB in different biofluids after incubating with sealed 2‐PIMs. All values are expressed as mean ± SD, *n* = 3. From left to right: phosphate‐buffered saline (PBS), artificial cerebrospinal fluid (ACSF), simulated salivary fluid (SSF), simulated gastric fluid (SGF), simulated intestinal fluid (SIF), human plasma (Plasma), horse serum (Serum). Positive control: unsealed 2‐PIM in PBS (Unsealed).

To seal the microrobot with a thin yet strongly adhered layer of NIR‐responsive PCL diol, we employed a dip‐sealing process (**Figure** **3**a). Briefly, following RhB loading into the chamber, the PIMs were brought into direct contact with spin‐coated PCL diol films, which then rapidly separated from the substrate, cooled down, and formed sealing layers over the pores. To achieve the required level of precise control of time and force, a mechanical sensor was utilized to produce transient contact and monitor force during the process. Successful sealing required physical contact between the PIMs and PCL diol film, which was visually verified by the presence of red‐colored stamps on the sealing substrate caused by the physical transfer of RhB during successful contact. The morphologies of the PIMs before and after sealing were further studied by bright‐field microscopy and SEM (Figure 3b). 1‐PIMs and 2‐PIMs showed uniform and dense sealing layers that were able to fully cover the pores, while the larger exposure pores of 3‐PIMs were more difficult to fully cover. Furthermore, all three kinds of PIMs demonstrated complete structural integrity with no visible signs of damage, verifying the feasibility of the sealing process using controlled mechanical contact. The cylindrical internal geometry was integral to supporting the structural integrity of the PIMs during this process, as demonstrated through comparisons with thin‐walled shell designs in earlier experiments that failed to retain structural integrity during dip‐sealing (Figure S11, Supporting Information). Thus, the PIMs with the cylindrical internal cavity geometry were chosen in this study. Moreover, all three kinds of PIMs demonstrated much lower drug leakage compared with unsealed controls, confirming strong drug protection provided by the sealing layers (Figure 3c). The effect of different pore designs on drug leakage was further studied through long‐term incubation of sealed PIMs (Figure 3d). Similar to the results observed by SEM images, 1‐PIMs and 2‐PIMs showed robust drug protection with low drug leakage ratios (4.04% ± 1.24% for 1‐PIMs and 4.15% ± 2.79% for 2‐PIMs, *p* = 0.929146 for 1‐PIMs vs 2‐PIMs), while 3‐PIMs showed significantly higher drug leakage ratios at 9.86% ± 1.72% (^****^ *p* = 0.000051 for 1‐PIMs vs 3‐PIMs, ^**^ *p* = 0.001655 for 2‐PIMs vs 3‐PIMs). As 2‐PIMs showed higher drug loading capacity compared to 1‐PIMs and stronger drug protection compared to 3‐PIMs, this design was chosen as the optimized PIM type for further studies. The drug protection ability of 2‐PIMs was further investigated in different biofluids representing various drug delivery target sites, such as gastrointestinal administration and blood circulation administration (Figure 3e). In each biofluid, the fluorescence intensity of RhB was significantly lower after incubating with sealed PIMs compared with unsealed PIMs, demonstrating superior drug protection facilitated by the design features of 2‐PIMs.

### 2.4 Drug Release of the PIMs

The controllable on‐demand release of therapeutics is another significant consideration when designing microrobotic drug delivery systems, which can be defined from two different perspectives: on‐demand burst release and on‐demand pulsed release. For the on‐demand burst release which is focused on in this paper, previous studies have demonstrated microrobots with various mechanisms for controlled on‐demand payload release including ultrasound,^[^ ^25a^ ^]^ UV radiation,^[^ ^18^, ^19^, ^30^ ^]^ pH changes,^[^ ^21^, ^26^, ^38^ ^]^ and NIR radiation.^[^ ^11^, ^39^ ^]^ Among them, NIR radiation possesses a combination of advantages that make it ideal for this work, including noninvasiveness, remarkable penetration depth, high translational potential, and flexible spatiotemporal specificity. However, the on‐demand trigger of drug release by NIR radiation is normally achieved by cumbersome processing steps requiring chemical modification of drugs with photocleavable linkers and grafting drugs onto the microrobots. Our puffball‐inspired microrobot drug delivery system benefits from the sealing layer on the PIM which also functions as the triggerable drug‐release mechanism. Through appropriate modification of the sealing layer rather than through direct modification of the drugs themselves, we can confer NIR‐responsive properties. Our system therefore provides more freedom in the variety of loadable drugs than previously reported microrobots,^[^ ^11^, ^39^ ^]^ making it a versatile platform for drug delivery applications. For the on‐demand pulsed release, an interesting feature of the PIM system is that the sealing layer may re‐solidify after the NIR‐trigger melt, which provides the potential to regulate the drug release rate by switching on/off the NIR laser.

To achieve the desired on‐demand trigger, we investigated a range of photothermal agents (Table S2, Supporting Information) and chose IR‐780 iodide, a lipophilic cationic near‐infrared dye that is widely used in photothermal therapy and photodynamic therapy. IR780 is ideal for this study due to its superior biocompatibility, excellent miscibility with PCL diol, and outstanding photothermal ability.^[^ ^40^ ^]^ Furthermore, it has been reported that the optical stability of IR780 can be enhanced through encapsulation into phase change materials to reduce photo‐oxidative bleaching.^[^ ^41^ ^]^

**Figure 4:**
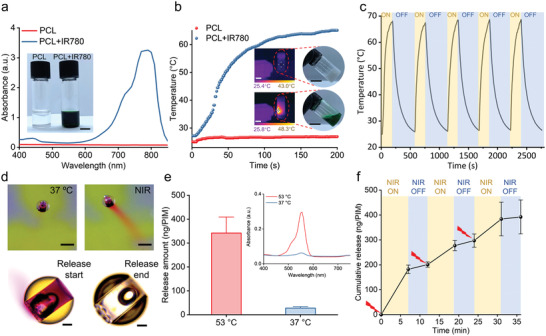
Drug release capability of the microrobotic system. a) UV–vis–NIR absorption spectra of PCL and PCL‐IR780. The inserted images show the PCL diol and PCL diol with IR780. Scale bar = 1 mm. b) Photothermal heating curves of PCL and PCL‐IR780 under NIR laser irradiation (808 nm, 0.6 W cm^−2^) over 200 s. The inset images are infrared thermal images under laser exposure (left) and photos after irradiation (right) of PCL (top) and PCL‐IR780 (bottom). Scale bars = 2 cm. c) Temperature variation of PCL‐IR780 under five cycles of NIR laser irradiation and cooling. d) Top: Photos of 2‐PIM at 37 °C (left) and after NIR laser irradiation (right). Scale bars = 300 µm. Bottom: Bright‐field microscopy images of the 2‐PIM before (left) and after (right) release. Scale bar = 50 µm. e) The release amount of RhB after long‐term incubation at 37 and 53 °C. All values are expressed as mean ± SD, *n* = 6. Inset figure shows the UV–Vis spectra of the solutions after incubating the PIMs at 37 and 53 °C. f) On‐demand release of RhB from 2‐PIMs under the triggering of on/off switching NIR laser. All values are expressed as mean ± SD, *n* = 3.

After blending PCL diol with IR780 (PCL‐IR780), a change in the solution from colorless to green indicated successful mixing of IR‐780 molecules (**Figure** **4**a and Figure S12, Supporting Information). This was further confirmed using UV–vis–NIR absorption spectra, where pristine PCL diol showed no absorbance across all wavelengths, while PCL‐IR780 exhibited strong absorption around the NIR region, indicating potential for photothermal conversion.^[^ ^40a^ ^]^ Additionally, the absorption characteristic peak of IR780 showed a slight red shift toward 800 nm in PCL‐IR780, which might be ascribed to the aggregation of IR780 and renders it excitably by the common 808 nm NIR laser.^[^ ^41^ ^]^ The photothermal characteristics of PCL and PCL‐IR780 were further investigated by monitoring the temperature profile under a NIR laser at 808 nm and the physical process under an IR thermal camera (Figure 4b and Video S1, Supporting Information). Upon exposure to laser irradiation, the temperature of PCL‐IR780 increased rapidly to 65.1 °C in 200 s, while the temperature of PCL diol remained unchanged, confirming successful photothermal conversion. Importantly, this temperature increase also melted the PCL sealing layer, which was visually confirmed in both IR thermal images and photos, further verifying the photothermal properties of the sealing layer and its potential for on‐demand drug release. The stability of the photothermal effect was also investigated by pulsed “on–off” NIR irradiation, with five cycles of irradiation and cooling (Figure 4c). The amplitude of temperature variation relative to the amount of irradiation was consistent, indicating photothermal stability of PCL/IR780 without overheating or photobleaching. Despite the elevated temperature from NIR irradiation, the risk of tissue damage caused by the photothermal triggering remains low. Compared to other microrobotic systems where the entire microrobot body is NIR‐responsive,^[^ ^11^, ^39^ ^]^ PIMs are designed with NIR responsiveness only on the sealing cap, leading to a very small heating area requiring reduced heating time, thereby reducing the risks of photothermal‐related potential tissue damage. Moreover, the reduced heat sensitive area enables short exposure times with remaining heat quickly absorbed by the surrounding physiological fluids (Figure S13, Supporting Information), which further minimizes any potential damage to surrounding tissues.

Although it is known that NIR light may degrade when penetrating deep tissue, a high concentration of IR780 in the sealing layer can improve the NIR‐sensitivity. To demonstrate this, pig skin was employed to emulate a biological tissue barrier against NIR light (808 nm, 0.6 W cm^−2^) used to irradiate PCL/IR780 formulations containing different concentrations of IR780 (0.1 and 10 mg mL^−1^) (Figure S14, Supporting Information). Under these conditions, the temperature of PCL with 0.1 mg mL^−1^ IR780 only increased slightly and failed to melt the PCL due to NIR degradation through the pig skin, whereas PCL with 10 mg mL^−1^ IR780 showed a significant photothermal response that successfully melted the PCL. To further improve the photothermal ability of the sealing layer on PIMs, PCL with an ultrahigh concentration of IR780 (50 mg mL^−1^) was prepared to seal the PIMs, and tetrahydrofuran (THF) was employed as a cosolvent that became evaporated during the sealing process. Then the controllable drug‐release of PIMs under NIR irradiation was studied. No release or leakage of RhB from PIMs was observable at 37 °C, while red clouds of RhB emerged from the PIMs once exposed to NIR laser, demonstrating similar payload release characteristics to puffballs (Figure 4d and Video S2, Supporting Information). Bright‐field microscopy further confirmed successful payload release, where complete RhB release under NIR irradiation was verified by the red cloud surrounding PIMs and the subsequent empty internal chamber. We also studied the cumulative release profile of the PIMs at 37 and 53 °C to test the effectiveness of the sealing layer under different temperatures (Figure S15, Supporting Information). Over the course of 120 min, small amounts of RhB were released when incubated at 37 °C, whereas significantly higher amounts of RhB were released when incubated at 53 °C. It is hypothesized that the observed RhB release at 37 °C was due to free RhB attached to the external body of PIMs, as previous data confirmed RhB payload trapped within the internal chamber was protected by the sealing layer. Therefore, the quantity of RhB released from the PIMs at 37 or 53 °C under a long‐term incubation (24 h) was quantified (Figure 4e). The PIMs show negligible release at 37 °C (27.61 ± 4.15 ng per PIM) with a low absorbance peak, while incubation at 53 °C significantly increased RhB release (341.74 ± 44.84 ng per PIM) due to the gradual melting of the sealing layer, confirming the high thermal‐sensitivity of the sealing layer design.

Furthermore, we demonstrated the ability for PIMs to exhibit pulsed release under NIR‐triggered on/off switching cycles (Figure 4f). Initial RhB release was observed after NIR irradiation, due to the required temperature increase to melt the sealing layer. Upon switching NIR off, RhB release decreased significantly as the sealing layer solidified and re‐sealed the pores. Although SEM images of the PIMs after one NIR on/off cycle show that some pores remained partially exposed, which results in a slow gradual release of cargo (Figure S16, Supporting Information), the bulk cargo release rate was controllable using NIR‐triggered on/off switching cycles, demonstrating the robust control of PIMs over drug release and their potential for smart dosing therapeutics. Additionally, to study the potential of PIMs for large‐scale preparation, a 10 × 10 array of PIMs was printed and loaded using the same strategy (Figure S17, Supporting Information). All the PIMs are successfully loaded with RhB solution (as evidenced by the observation of red RhB solution within PIMs), while burst release of RhB from the PIMs after triggering was observed. These results highlight that the PIMs have great potential in scaled‐up industrial translational processes.

### 2.5 Locomotion, Targeting, and On‐Demand Drug Delivery of the PIMs

**Figure 5:**
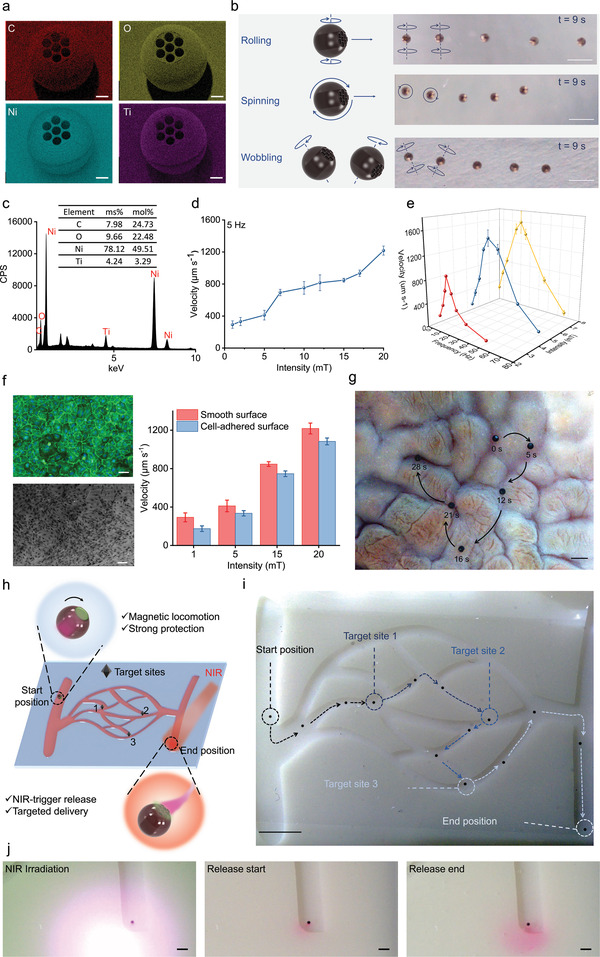
Locomotion, targeting, and on‐demand drug delivery of the microrobotic system. a) EDX mapping of the PIMs after Ni/Ti physical vapor deposition. Red: carbon, yellow: oxygen, cyan: nickel, purple: titanium. Scale bars = 50 µm. b) Schematic of superimposed snapshots of PIMs following three different motion modes under a rotating magnetic field. The PIMs were observed from a top view. Scale bars = 500 µm. c) EDS of PIMs after Ni/Ti deposition showing mass fractions (wt%) and atom fractions (at%) of C, O, Ni, and Ti. d) PIM motion velocity under different rotating magnetic field intensities (1–20 mT, frequency fixed at 5 Hz). All values are expressed as mean ± SD, *n* = 3. e) PIM motion velocity at different rotating magnetic field frequencies (1–70 Hz, intensity fixed at 2.5, 5, and 7.5 mT). All values are expressed as mean ± SD, *n* = 3. f) The CD31/DAPI staining confocal image (upper left, scale bar = 50 µm) and SEM image (lower left, scale bar = 100 µm) of the cell‐adhered surface. The velocities of PIMs on cell‐adhered surfaces versus smooth surfaces under different rotating magnetic field intensities (5 Hz, 1–20 mT). All values are expressed as mean ± SD, *n* = 3. g) Snapshots of the motion of PIMs on an ex vivo pig stomach. Scale bar = 1 mm. h) Schematic of the on‐demand targeted delivery of cargos by PIMs in a 3D‐printed chip with vessel‐like fluidic channels. i) Controlled locomotion of the PIM along the designated trajectory under the rotating magnetic field in the sinuous channels. Scale bar = 5 mm. j) Images of the end position with NIR laser: after application of NIR laser, a red cloud of RhB cargo was delivered to the targeted area. Scale bars = 1 mm.

Magnetic actuation through low‐strength high‐penetration magnetic fields that are noninvasive and harmless to the human body is one of the most promising and popular strategies for controlling microrobots due to its localization precision, free of fuel requirements, robust handling, and biocompatibility.^[^ ^36a^ ^]^ During fabrication, physical vapor deposition (PVD) was used to first deposit nickel (Ni) layers onto the surface of the PIMs to enable remote manipulation and actuation using low‐strength magnetic fields, and then deposit titanium (Ti) layers to ensure biocompatibility and prevent Ni oxidation (see Figure 1c).^[^ ^42^ ^]^ It has been reported that Ni/Ti composite layers on 3D‐printed micromachines do not induce cytotoxicity and can promote cell proliferation.^[^ ^43^ ^]^ We performed in vitro cytotoxicity studies to investigate the cell viability of fibroblast cells on the PIMs after 24 h culture. The relative high frequency of green and low frequency of red cells in the LIVE/DEAD results showed that cell viability was unaffected in the presence of PIMs (Figure S18a, Supporting Information). Quantification of the AlamarBlue assay results in Figure S18b (Supporting Information) revealed no significant difference in Ni/Ti coated PIMs (94.34 ± 2.94%), noncoated PIMs (98.26 ± 2.25%), and untreated cells (100%). After confirming the biocompatibility, qualitative evaluation of the distribution and composition of Ni and Ti performed with energy‐dispersive X‐ray (EDX) mapping showed homogeneous distribution of elements on the surface of the PIMs from both the top view and tilted view, indicating the successful deposition of Ni/Ti composite layers (**Figure** **5**a and Figure S19, Supporting Information). Energy dispersive spectra (EDS) further verified a sufficiently high content of nickel on the surface, which ensured high magnetic responsiveness of the PIMs (Figure 5c).

We then assessed the locomotion behavior and performance of the PIMs under a magnetic field in aqueous media. Although gradient magnetic fields can actuate microrobots,^[^ ^44^ ^]^ their jerky pulling motion has been shown to lower locomotive precision.^[^ ^36a^ ^]^ Therefore, an external magnetic actuation (EMA) system composed of eight electromagnetic actuator coils (MFG‐100; Magnebotix, Zurich, Switzerland) was employed to generate a rotating magnetic field to actuate and steer the PIMs by exerting a rotational torque. Under this rotating magnetic field, the PIMs demonstrated rapid magnetic response and could convert the magnetic torque into directional movement as the neighboring hydrodynamic boundary led to the mismatching of hydrodynamic mobility around the PIMs.^[^ ^19^, ^26^, ^45^ ^]^ Interestingly, the PIMs presented three different modes of motion in response to varying rotating magnetic field inputs: i) a rolling motion whereby the PIM exhibited a rotational axis parallel to the frictional surface and perpendicular to its direction of motion; ii) a spinning motion whereby the PIM exhibited a rotational axis perpendicular to both the frictional surface and its direction of motion; iii) a wobbling motion whereby the PIM exhibited a combination of rolling and spinning motions (Figure 5b and Video S3, Supporting Information, the model PIM was observed from a top view). Similar to a previous study,^[^ ^46^ ^]^ the rolling mode showed the highest velocity while the spinning mode showed the lowest, indicating that PIMs can be velocity‐modulated using different motion modes according to various application scenarios. The velocity increased by increasing the strength of the rotating magnetic field, reaching a translational motion speed of up to 1217.99 ± 56.52 µm s^−1^ (Figure 5d). The velocities of the PIMs were evaluated as a function of the rotating magnetic field (RMF) frequency at magnetic field intensities of 2.5, 5, and 7.5 mT (Figure 5e). The overall velocities of PIMs increased almost linearly with increasing the applied magnetic field frequency at all magnetic intensities. There existed a certain RMF frequency known as step‐out frequency, at which the maximum forward velocity was achieved. When operating above the step‐out frequency, the velocities of the PIMs dramatically decreased and the locomotion of the PIMs became defective (moving unstably in random directions). This loss of velocity and control was due to desynchronization between the rotating magnetic field and the PIMs such that the PIMs were no longer able to overcome the resistive torque exerted by the fluid.^[^ ^24a^ ^]^ The step‐out frequency is related to intrinsic properties of the microrobot including the magnetization of the microrobot, drag force, friction, and field intensity, and it increases with increasing the field intensity.

In order to further probe the magnetic motion of these PIMs, we tested the limits of magnetically‐driven motion over rough surfaces as a robust demonstration of their capabilities. Human umbilical vein endothelial cells (HUVEC) were seeded on glass surfaces and confirmed by both CD31/DAPI staining and SEM images (Figure S20, Supporting Information). The cells were then fixed and PIMs were manipulated on the cell‐adhered surfaces under rotating magnetic fields with a frequency of 5 Hz and different intensities (1–20 mT). The velocities were measured and compared with the results of the smooth surfaces. As shown in Figure 5f, the velocity of the PIMs on cell‐adhered surfaces decreased by 40% compared with smooth surfaces under 1 mT and 5 Hz rotating magnetic fields, which could be ascribed to the increased friction. However, when the magnetic field intensity increased to 20 mT, the PIMs manipulated on cell‐adhered surfaces only experienced an 11% reduction in velocity compared to the smooth surfaces, reaching a translational motion speed of 1083.42 ± 35.49 µm s^−1^. Furthermore, we demonstrated precise control of the direction of PIMs along the trajectories by adjusting the direction of the rotating magnetic field. The PIMs were initially manipulated and showed controlled locomotion along the desired trajectories of various basic shapes (triangle, rectangle, and rhombus) (Figure S21 and Video S4, Supporting Information). Moreover, an ex vivo demonstration of manipulating the PIMs was performed using a pig stomach which presented a complex path due to the gastric rugae of the internal surface. As shown in Figure 5g, the PIM shows excellent fidelity to the designated path, demonstrating their capacity for precise navigation in targeted drug delivery applications.

Finally, we demonstrated the feasibility of the PIMs for targeting and on‐demand drug delivery in an in vitro environment designed to recapitulate in vivo features—namely, a 3D‐printed chip with vessel‐like fluidic channels—exposed to a low‐strength rotating magnetic field. The PIM was firstly loaded with RhB, sealed with the NIR‐responsive sealing layer, and then maneuvered from the starting position to three designated target positions (Figure 5h). Upon reaching the target position, an NIR laser was focused on the site to trigger melting of the sealing layer and subsequent release of the payload. Robust actuation and precise navigation of the PIM were implemented through diverse branching paths in the channel with no observable payload leakage, indicating the strong protection of payload during the moving process (Figure 5i and Video S5, Supporting Information). It was observed that the PIM sometimes collided with the sidewall as it was manually controlled in a low magnification vision which led to the overcorrection of its direction. This might be addressed by integrating closed‐loop control on the PIMs in future work which could achieve timely feedback. After NIR irradiation, a large amount of RhB diffused around the targeted area, indicating successful drug distribution (Figure 5j). Together, these results demonstrate the PIM's robust drug protection, precise locomotion, and on‐demand delivery properties, thereby confirming its potential for targeted drug delivery.

Additionally, although the PIMs were clearly visible throughout in vitro demonstrations, they cannot be directly visualized by optical microscopy during in vivo applications due to the low penetrating capacity of light. Therefore, the real‐time imaging properties of PIMs were also studied to show their imaging potential using X‐ray‐based imaging and fluorescence‐based imaging techniques (Figure S22, Supporting Information). As shown in Figure S22a (Supporting Information), high‐resolution images of the structure and internal chamber of the PIM were obtained under X‐ray imaging at different magnification times, and the contrast between the PIM and the surrounding was high due to the Ni/Ti coating. These results illustrated that the proposed PIM system has potential for applications in X‐ray real‐time imaging. Figure S22b (Supporting Information) showed the fluorescence molecular tomography (FMT) results under two wavelengths (635 and 790 nm). The PIMs could be clearly imaged under both wavelengths since many components exhibited fluorescence emission, while the signal intensity under 790 nm was remarkably stronger which could be ascribed to the great fluorescence efficiency of IR780. All the four PIMs injected could be easily discerned with high spatial clarity, therefore demonstrating their excellent fluorescence imaging capabilities. Furthermore, the proposed PIM system offered high flexibility in the loaded cargoes, sealants, and metallic coatings, thereby providing a rich choice of contrast agents (such as dyes and quantum dots for fluorescence imaging, lipiodol for X‐ray imaging, gold and indocyanine green for optoacoustic imaging) which could be integrated to potentially increase the sensitivity of imaging and provide multimodal imaging abilities.

## 3 Conclusion

To address the unmet challenges of microrobotic DDSs including large cargo loading, strong cargo protection, and on‐demand cargo release, we developed a bioinspired magnetic microrobotic system for drug delivery that replicates the unique characteristics of puffball fungi (like high spore payloads, protection of payload by the barrier cap and spheroidal fruitbody, and payload release following environmental stimulus of the barrier cap). The PIM was composed of a spherical shape coated in Ni/Ti for propulsion, a spatially separated internal chamber for drug loading, and a NIR‐responsive sealing layer for payload protection. Three kinds of PIMs with different lid designs were fabricated by two‐photon‐based 3D microfabrication. The fluorescent model cargo RhB was selectively loaded inside the chamber at high quantities dependent on the lid design and initial loading concentration. An automated dip‐sealing method was proposed to construct the NIR‐responsive sealing layer, which showed strong drug protection under various biological environments and robust photothermal abilities. The release of RhB was confirmed to possess on‐demand controllability through NIR laser irradiation. The PIMs could be efficiently actuated and steered under a rotating magnetic field, showing outstanding moving velocity which could be modulated by controlling three different motion modes. The benefits of robust drug protection, precise locomotion, and on‐demand delivery were demonstrated in a simulated in vitro environment using a 3D‐printed chip with vessel‐like fluidic channels. The PIMs described here provide a bioinspired strategy to construct an advanced microrobotic system with unique features and potential for targeted drug delivery applications.

## 4 Experimental Section

### Materials

Propylene glycol monomethyl ether acetate (PGMEA, ReagentPlus, ≥99.5%), 3‐(trimethoxysilyl)propyl methacrylate (Silane A174, 98%), rhodamine B (RhB, 95%), polycaprolactone diol (PCL diol, average *M_n_* ≈2000), IR‐780 iodide (≥95%), potassium chloride (KCl, ≥99%), calcium chloride (CaCl_2_, ≥93.0%), 4‐(2‐hydroxyethyl)piperazine‐1‐ethanesulfonic acid (HEPES, ≥99.5%), d‐glucose (≥99.5%), hydrochloric acid (HCl), phosphate buffered saline (PBS), potassium phosphate monobasic (KH_2_PO_4_, ≥99%), sodium bicarbonate (NaHCO_3_, Hybri‐Max, ≥99.5%), magnesium chloride hexahydrate (≥99%), ammonium bicarbonate ((NH_4_)HCO_3_, ≥99.5%), poly(ethylene glycol) 400 (PEG 400), agarose, and horse serum were obtained from Sigma‐Aldrich. IP‐S photoresist was purchased from Nanoscribe GmbH. SYLGARD 184 Elastomer Kit, sodium chloride (NaCl, ≥99.9%), sodium hydroxide (NaOH, ≥98.5%), ethanol absolute (≥99.9%), dimethyl sulfoxide (DMSO, ≥99.7%), and tetrahydrofuran (THF, ≥99.9%) were obtained from VWR International Ltd. Isopropanol (IPA, ≥99%) was purchased from Fisher Chemical. Polycaprolactone diol (PCL diol, average *M*_w_ ≈525–575) was purchased from Biosynth Carbosynth. Anti‐Adherence Rinsing Solution was purchased from STEMCELL Technologies. PrimaCreator White Value UV resin was purchased from Prusa Research. Dulbecco's Modified Eagle's Medium (DMEM), fetal bovine serum (FBS), and penstrep were obtained from Gibco, Thermo Fisher Scientific. AlamarBlue Cell Viability Reagent and LIVE/DEAD viability/cytotoxicity Kit were obtained from Invitrogen, Thermo Fisher Scientific. Fresh whole human blood (containing sodium citrate anticoagulant) was obtained from Cambridge Bioscience via the Imperial College Healthcare Tissue Bank (ICHTB). ICHTB is supported by the National Institute for Health Research (NIHR) Biomedical Research Centre based at Imperial College Healthcare NHS Trust and Imperial College London. ICHTB is approved by Wales REC3 to release human material for research (17/WA/0161). Ultrapure water was obtained from a Triple Red Avidity Science Duo at 18.2 mΩ cm^−1^. All the chemicals were used as received.

### PIM Fabrication

The structures of the microrobots were designed using computer assisted drawing software (Autodesk Inventor, Version 2020), and the final printing files were obtained using Describe (v2.5.5, Nanoscribe GmbH). ITO‐coated glass slide substrates (Nanoscribe GmbH) were first treated with oxygen plasma (1.0 mBar, 100% power) for 5 min (Plasma Prep5, Gala Instrument) and then immersed into Silane A174 solution (0.5% v/v in ethanol) to promote adhesion. Photoresist IP‐S resin was dropped on to the treated substrate and then transferred to the commercial two‐photon (2PP) 3D printing system (Photonic Professional, Nanoscribe GmbH, Germany) using a 25× immersion objective. Once printing was completed, the microrobots on the substrate were immersed into photoresist solvent PGMEA for 30 min followed by IPA for 30 min to remove unreacted resin. Samples were subsequently photocured under 405 nm light for 2 h and stored dry at room temperature for further use. For physical vapor deposition of Ni/Ti layers, PIMs were carefully dried with N_2_ flow and then sequentially sputtered with Ni (800 nm) and Ti (20 nm) nanofilms using a sputter deposition system (Hex Thin Film Deposition system, Korvus Technology, United Kingdom). The PIMs were premagnetized under a 1.8 T magnetic field to orient the magnetization direction after fabrication and before performing motion experiments.

### Characterization and Imaging of the PIMs

To assess the structural and morphological features, the PIMS were completely dried, sputtered coated (Au, 200 Å), and imaged by scanning electron microscopy (JEOL 6010LA). Energy‐dispersive X‐ray spectroscopy (EDX mapping) analysis and energy‐dispersive spectroscopy (EDS) were also performed to investigate the distribution and content of elements (C, O, Ni, and Ti) on the PIMs.

### Drug Loading

Drug loading was achieved using a centrifugation‐based physical loading method to facilitate drug payload ingress through the pores at the top of the structure. PIMs adhered onto glass substrates were first treated by oxygen plasma (1.0 mBar, 100% power) for 5 min to enhance hydrophilicity (Plasma Prep5, Gala Instrument). A ring‐shaped poly(dimethylsiloxane) (PDMS) mold, used to hold the loading solution, was prepared by mixing the SYLGARD 184 elastomer and curing agent to a ratio of 10:1, curing at 60 °C for 1 h, and hole‐punched using a hollow pore punch (JLB320CM, Boehm). After fixing the ring‐shaped PDMS mold onto substrates surrounding the attached PIMs, RhB solutions with 1–3% (w/v) concentrations were placed on top of the plasma‐treated PIMs and loaded via centrifugation at 859 rcf for 5 min at room temperature (Centrifuge 5910R, Eppendorf). After centrifugation, excess RhB solution was discarded and the loaded PIMs air‐dried under N_2_ flow. This entire process was repeated six times to reach loading capacity. After the final loading process, residual RhB solution covering the substrate was removed using a pipette with residues blot‐removed using tissue paper. This prevented the excess cargo solution from mixing with PCL during the subsequent sealing step. The loaded PIMs were stored at −20 °C for the next step. To confirm cargo loading into the internal chamber structure, loaded PIMs were first sealed to prevent inaccuracies resulting from cargo evaporation, and then imaged under bright‐field microscopy using 10× and 20× objectives (EVOS XL core), fluorescence microscopy (Zeiss Axio Observer Z1 microscope) and confocal laser scanning microscopy (Leica SP8 confocal microscope). To assess the loading volume, PIMs without bottom layers were printed and loaded using the same procedure. After careful wash, the loaded PIMs without bottom layers were removed from the substrate and conduct quantitative analysis based on standard curve absorbance spectroscopic analysis (Supporting Information). PEG and DMSO were also loaded into the PIMs to demonstrate their versatility. 0.5% (w/v) RhB/PEG 400 solution and 2% (w/v) RhB/DMSO solution were prepared and loaded into the PIMs using the same centrifugation procedure. After loading and sealing, the PIMs were imaged under bright‐field microscopy (EVOS XL core).

### Dip‐Sealing

Melting temperature of PCL diol was determined by assessing the viscosity‐temperature curve through a rheometer (MCR302 Modular Compact Rheometer, Anton Paar). To prepare the sealing layer, PCL diol *M*_w_ 525–575 and PCL diol *M*_n_ 2000 were first fully melted at 70 °C and mixed to a ratio of 2:1 w/w via 5 min vortex. IR780/THF solution was then added to a final concentration of 5 wt% and thoroughly mixed via 5 min vortex and 30 min ultrasound sonication. The PCL/IR780 sealing solution was then spin‐coated onto a plasma‐treated glass slide at 1000 rpm for 30 s with an acceleration speed of 500 rpm using a spin‐coater (Model WS‐650SZ‐6NPP/Lite, Laurell technologies corporation). To dip seal the PIMs, the contact function of the rheometer (MCR302 Modular Compact Rheometer, Anton Paar) was employed whereby PIMs were mechanically and controllably brought into contact with the sealing glass slide three times. Sealed PIMs were rinsed thoroughly with ultrapure water and ethanol before use to remove excess cargo on the outside of the microrobots. The sealing quality was evaluated using different pore designs and different media solutions (Supporting Information).

### Photothermal Properties of Sealing Material

The UV–vis–NIR absorption spectra of PCL and PCL‐IR780 were obtained using a plate reader (Molecular Devices Spectramax M5) between wavelength 400–850 nm with a 10 nm step. To obtain the photothermal heating curves, PCL with or without IR780 was irradiated with a NIR laser (808 nm, 0.6 W cm^−2^) where laser power was determined by a digital handheld optical power and energy meter console (PM100D, Thorlab GmbH, Dachau Germany). A digital data logger thermometer (HH306A, Omega) and an IR thermal imaging camera (FLIR ONE Pro, Flir, USA) were employed to record the temperature variation and visually observe the process, respectively.

### Drug Release

Loaded and sealed PIMs were carefully removed from the substrate and transferred to ultrapure water. For temperature‐induced release, PIMs were heated to 37 and 53 °C, and the release amounts at different time points were quantified by UV–Vis spectroscopy (Molecular Devices Spectramax M5). For NIR‐trigger release, loaded RhB was released from PIMs by irradiating under NIR for 2 min followed by 5 min of standing time. For pulsed drug release, this operation was repeated for 3 cycles with 5 min in between each cycle, and the released amount quantified after each cycle.

### Magnetic Control

PIMs were magnetically actuated using an EMA system composed of eight electromagnetic actuator coils (MFG‐100; Magnebotix, Zurich, Switzerland), which could generate variable magnetic fields (field range of 0–20 mT) with three degrees of freedom in position (*x*, *y*, and *z*) and two degrees of freedom in orientation (yaw and roll). A puA2500‐14uc charge‐coupled device camera (Basler pulse) is employed to record the videos with constant capture settings including zoom and orientation. The swimming performance of PIMs was evaluated by actuating them three times under different rotating magnetic field parameters. PIM motion velocities were extracted from the video recordings using the motion tracking function of ImageJ (Fiji, ImageJ 1.53f51, National Institutes of Health, USA). A rotating magnetic field with 2 mT and 5 Hz was used to actuate the PIMs along programmable trajectories. The motion trajectories of the microbots were analyzed using basic motion detection and tracking algorithm, which was implemented in Python using open‐source Python packages. To study the motion on cell‐adhered surfaces, glass slides were autoclaved and seeded with human umbilical vein endothelial cells (HUVECs, 7 × 10^5^ cells). After culturing for 7 days, the cell layers were fixed with 4% paraformaldehyde solution and then applied to study the motion of PIMs. Rotating magnetic fields with 5 Hz and different intensities were used to actuate the PIMs, and the velocities were studied as previously described. For the ex vivo demonstration of motion, the PIMs were manipulated on pig stomach tissue (obtained from a local abattoir) with the rotating magnetic field of 20 mT and 5 Hz.

### Simulated in Vitro Demonstration of Targeted Drug Delivery

A chip with vessel‐like fluidic channels was 3D printed (Prusa SL1S Speed, Prusa Research) using the PrimaCreator White Value UV resin (Prusa Research). The finished print was washed with IPA, ultrasound sonicated for 10 min, and photo cured under 405 nm light for 2 h at 60 °C. The loaded and sealed PIM was carefully removed from the substrate and transferred into the chip. The PIM was steered under a rotating magnetic field with 2 mT and 5 Hz along the designated trajectory. Upon reaching the targeted area, a NIR laser (808 nm, 0.6 W cm^−2^) was applied to the PIM to trigger drug release. Videos were recorded by a puA2500‐14uc charge‐coupled device camera. The motion trajectories of the microbots were analyzed using the same methods as above.

### Statistical Analysis

All statistical analyses were performed using the IBM Statistical Package for the Social Sciences (v.26, SPSS, Chicago, IL, USA). Results were expressed as the mean ± standard deviation. All statistical comparisons were made using an unpaired, two‐tailed Student's *t*‐test. A difference with a *p*‐value of less than 0.05 was considered statistically significant. “ns” represents not significant, *p* > 0.05; *p ≤ 0.05, ^**^ *p* ≤ 0.01, ^***^ *p* ≤ 0.001, ^****^ *p* ≤ 0.0001.

## Conflict of Interest

The authors declare no conflict of interest.

## Author Contributions

X.S. and R.S. contributed equally to this work. M.M.S. supervised the project. X.S., R.S., and M.M.S. conceived and/or designed the research. X.S., R.S., and K.Z. designed and conducted the fabrication, loading, and sealing experiments of the PIMs. X.S. and R.S. designed and conducted the release and motion experiments of the PIMs. X.R. contributed to the motion experiments on cell‐adhered surfaces and X‐ray imaging experiments. J.L. contributed to biomimicking inspiration and conducted the cytotoxicity assay. D.G. wrote the scripts for motion video analysis of the PIMs. A.P. contributed to the ex vivo demonstration. R.Z. contributed to the LIVE/DEAD staining and fluorescent microscopy imaging. X.S., R.S., and R.W. contributed to the overall data analysis. All the authors discussed the results and assisted in the preparation of the paper. R.W. contributed to data visualization and figure design. X.S. drafted the manuscript. R.S., R.W., and M.M.S. revised the paper. All authors have given approval to the final version of the manuscript.

## Supporting information

Supporting Information

Supplemental Video 1

Supplemental Video 2

Supplemental Video 3

Supplemental Video 4

Supplemental Video 5

## Data Availability

The data that support the findings of this study are available from rdm‐enquiries@imperial.ac.uk upon reasonable request.
