# Supplementary material for: Puffball‐Inspired Microrobotic Systems with Robust Payload, Strong Protection, and Targeted Locomotion for On‐Demand Drug Delivery
Source: Adv Mater. 2022 Sep 27;34(43):2204791. doi: 10.1002/adma.202204791 (PMC11475404; doi:10.1002/adma.202204791)
Supplement: Supplementary file 1 — Supporting Information [file ADMA-34-2204791-s003.pdf]

ADVANCED MATERIALS

Supporting Information

for *Adv. Mater.,* DOI: 10.1002/adma.202204791

Puffball-Inspired Microrobotic Systems with Robust Payload, Strong Protection, and Targeted Locomotion for On-Demand Drug Delivery

*Xin Song, Rujie Sun, Richard Wang, Kun Zhou, Ruoxiao Xie, Junliang Lin, Dimitar Georgiev, Andrei-Alexandru Paraschiv, Ruibo Zhao, and Molly M. Stevens**

Supporting Information

Puffball-Inspired Microrobotic Systems with Robust Payload, Strong Protection, and Targeted Locomotion for On-Demand Drug Delivery

Xin Song,[a,#] *Rujie Sun,*[a,#] *Richard Wang,*[a] *Kun Zhou,*[a] *Ruoxiao Xie,*[a] *Junliang Lin,*[a] *Dimitar Georgiev,*[a, b] *Andrei-Alexandru Paraschiv,*[a,c] *Ruibo Zhao,*[a,d] *and Molly M. Stevens.*[a,]*

[#]These authors contributed equally to this work.

[a] Department of Materials, Department of Bioengineering, Institute of Biomedical Engineering, Imperial College London, London SW7 2AZ, UK
[b] Department of Computing, UKRI Centre for Doctoral Training in AI for Healthcare, Imperial College London, London SW7 2AZ, UK
[c] Department of Chemistry, Imperial College London, London SW7 2AZ, UK
[d] Institute of Smart Biomaterials, School of Materials Science and Engineering, Zhejiang Sci-Tech University, Hangzhou 310018, Zhejiang, China

[*]E-mail: m.stevens@imperial.ac.uk

Quantitative analysis of RhB.

Quantitative analysis of RhB was performed based on standard curve absorbance spectroscopic analysis. Briefly, a series of RhB aqueous solutions with different concentrations (5 µg/mL, 4 µg/mL, 3 µg/mL, 2 µg/mL, 1 µg/mL, 0.9 µg/mL, 0.8 µg/mL, 0.7 µg/mL, 0.6 µg/mL, 0.5 µg/mL, 0.4 µg/mL, 0.3 µg/mL, 0.2 µg/mL, 0.1 µg/mL) were prepared by diluting from a mother liquor (10 µg/mL) and then transferred into 96 half area well plate before measuring their absorbance at 553 nm using a plate reader (Molecular Devices Spectramax M5). The absorbance values for each concentration were averaged over 8 technical replicates and the standard curve was established fitting a least squares regression line to the absorbance values and concentrations (**Figure S5 and S6**): $y = 0.1626x + 0.0331$, where y represents absorbance value at 553 nm and x represents concentration (µg/mL). $R^2 = 0.9945$.

Quantification of PIMs loading

To evaluate the loading capacity of PIMs, specially designed PIMs without bottom layers were fabricated following the same procedures of the original PIMs using a Nanoscribe GmbH 2PP 3D printing system with a 25x immersion objective. These PIMs were loaded with RhB solution and sealed with PCL-IR780. The loaded and sealed PIMs were washed with ultrapure water and ethanol, and then carefully removed from the substrate into 200 µL ultrapure water in a 96-half area well plate. After 4-hour incubation, the PIMs were removed from the solution and the absorbance values were quantified at 553 nm using a plate reader (Molecular Devices Spectramax M5). The loading amounts of RhB in the PIMs were calculated based on the standard curve.

Evaluation of drug leakage

To study the drug protection of PIMs, the loaded and sealed PIMs were washed with ultrapure water and ethanol, and then carefully removed from the substrate into 200 µL ultrapure water in a 96-half area well plate. After 24-hour incubation, the PIMs were removed from the solutions and the absorbance values were quantified at 553 nm using a plate reader (Molecular Devices Spectramax M5) and the loading amounts of RhB in the PIMs calculated based on the standard curve. Specially designed PIMs without bottom layers were used as positive control. The drug leakage of loaded and sealed PIMs in different simulated biofluids was also studied. Artificial cerebrospinal fluid (ACSF)[1] was prepared using a composite solution of 0.14 mol/L NaCl, 0.005 mol/L KCl, 0.002 mol/L $CaCl_2$, 0.01 mol/L HEPES, and

0.01 mol/L D-glucose, and pH adjusted to 7.4. Simulated salivary fluid (SSF), simulated gastric fluid (SGF), and simulated intestinal fluid (SIF) were prepared following a standardized in vitro digestion environment.[2] Firstly, various electrolyte stock solutions were prepared: 37.3 g/L KCl, 68 g/L KH_2PO_4, 84 g/L $NaHCO_3$, 117 g/L NaCl, 30.5 g/L $MgCl_2(H2O)_6$ and 79 g/L (NH_4) HCO_3. SSF, SGF, and SIF were then prepared by mixing the stock solutions and adjusting the pH. SSF composed of 18.8 mmol/L K^+, 13.6 mmol/L Na^+, 19.5 mmol/L Cl^-, 3.7 mmol/L $H_2PO_4^-$, 13.7 mmol/L HCO_3^-, 0.15 mmol/L Mg^{2+}, 0.12 mmol/L NH_4^+, and 1.5 mmol/L Ca^{2+}, and then pH was adjusted to be 7. SGF composed of 7.8 mmol/L K^+, 72.2 mmol/L Na^+, 70.2 mmol/L Cl^-, 0.9 mmol/L $H_2PO_4^-$, 25.5 mmol/L HCO_3^-, 0.1 mmol/L Mg^{2+}, 1.0 mmol/L NH_4^+, and 0.15 mmol/L Ca^{2+}, and pH adjusted to 3. SIF composed of 7.6 mmol/L K^+, 123.4 mmol/L Na^+, 55.5 mmol/L Cl^-, 0.8 mmol/L $H_2PO_4^-$, 85 mmol/L HCO_3^-, 0.33 mmol/L Mg^{2+}, and 0.6 mmol/L Ca^{2+}, and pH adjusted to 8. Horse serum was obtained from Sigma-Aldrich, defrosted at 37 ºC and used directly. Human plasma was separated from human whole blood. Briefly, human fresh whole blood, from a healthy donor aged between 18-60, was collected using vacuum tubes containing sodium citrate as the anticoagulant and centrifuged at 2000g for 10 min at 4 ºC to obtain human plasma. Human blood samples were obtained from the Imperial College Healthcare Tissue Bank (ICHTB). ICHTB is supported by the National Institute for Health Research (NIHR) Biomedical Research Centre based at Imperial College Healthcare NHS Trust and Imperial College London. ICHTB is approved by Wales REC3 to release human material for research (17/WA/0161).

Cell viability of PIMs

Human pulmonary fibroblasts (HPF) cells were used as the model cell line for cell viability studies. The cells were expanded in T-75 cell culture flasks (Corning) containing Dulbecco's Modified Eagle's Medium (DMEM; Gibco™, Thermo Fisher Scientific) supplemented with 10% fetal bovine serum (Gibco™, Thermo Fisher Scientific) and 1% penstrep (Gibco™, Thermo Fisher Scientific). For the experiments, the cells were collected and seeded into a 96-well plate (HTS Transwell®-96 Tissue Culture Systems, Corning) at a concentration of 1×10^4 cells/well in triplicates. The respective PIM samples (PIMs with or without Ni/Ti coating, 2 microrobots for each group) were sterilized and added to the plate in triplicates. The samples were incubated for 24 hours and then removed followed by another 24-hour incubation. The cell viabilities were assessed using the AlamarBlue® Cell Viability Reagent (Invitrogen™, Thermo Fisher Scientific) according to the manual. LIVE/DEAD™

viability/cytotoxicity Kit (Invitrogen^{TM}, Thermo Fisher Scientific) was also employed to stain the live and dead cells before observed using a confocal laser scanning microscopy (Leica SP8 confocal microscope). The live cells were imaged at 494/517 nm, while the dead cells were imaged at 528/617 nm.

Imaging property of PIMs

X-ray-based imaging and fluorescence-based imaging techniques were employed to study the imaging property of PIMs. The PIMs were carefully removed from the substrate and then injected into 2 wt.% agarose solutions in a well plate that was coated with anti-adherence rinsing solution (STEMCELL Technologies, United Kingdom). After cooling at 4 °C for 30 minutes, the agarose hydrogels with PIMs were carefully removed, then applied to an X-ray tomography system (Xradia 510 Versa, Zeiss, Germany) for X-ray-based imaging and a fluorescence tomography system (FMT 4000, PerkinElmer, Inc., USA) for fluorescence-based imaging.

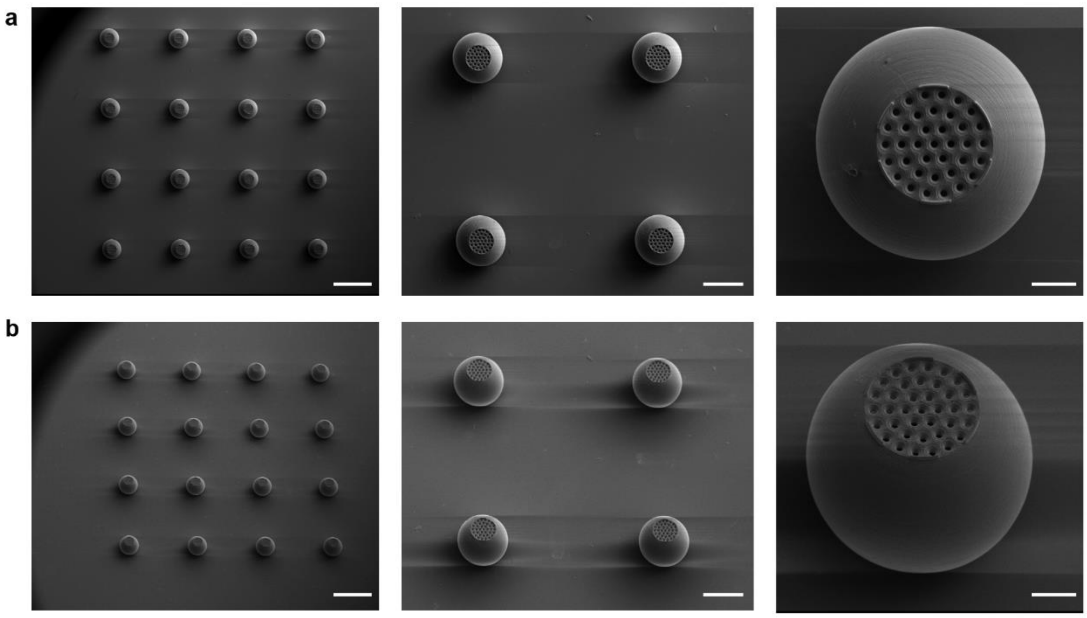

Figure S1. SEM images of the 1-PIMs from top view a) and tilted view b): (*left*) a 4×4 array of 1-PIMs on substrate, (*center*) a 2×2 array of 1-PIMs on substrate, (*right*) and a single 1-PIM on substrate. Scale bars from left to right, 500 μm, 200 μm, 50 μm.

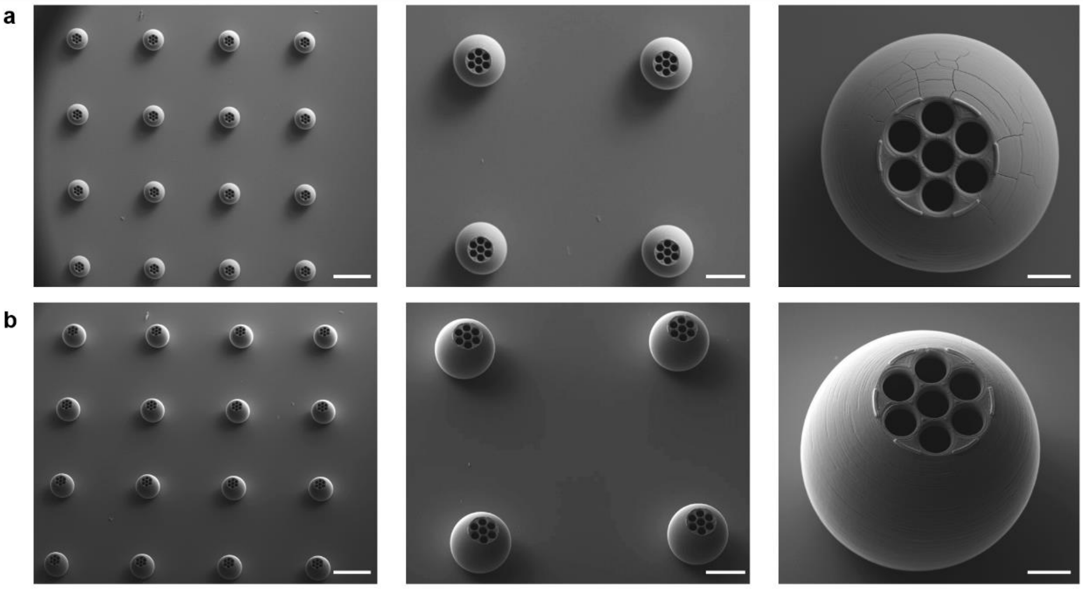

Figure S2. SEM images of the 2-PIMs from top view a) and tilted view b): (*left*) a 4×4 array of 2-PIMs on substrate, (*center*) a 2×2 array of 2-PIMs on substrate, (*right*) and a single 2-PIM on substrate. Scale bars from left to right, 500 μm, 200 μm, 50 μm.

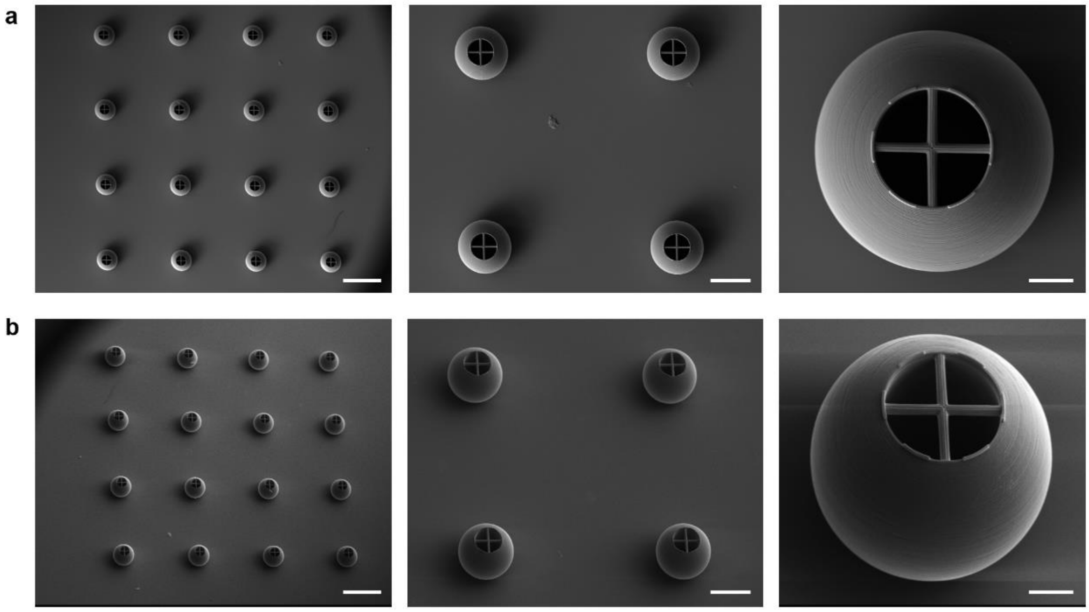

Figure S3. SEM images of the 3-PIMs from top view a) and tilted view b): (*left*) a 4×4 array of 3-PIMs on substrate, (*center*) a 2×2 array of 3-PIMs on substrate, (*right*) and a single 3-PIM on substrate. Scale bars from left to right, 500 μm, 200 μm, 50 μm.

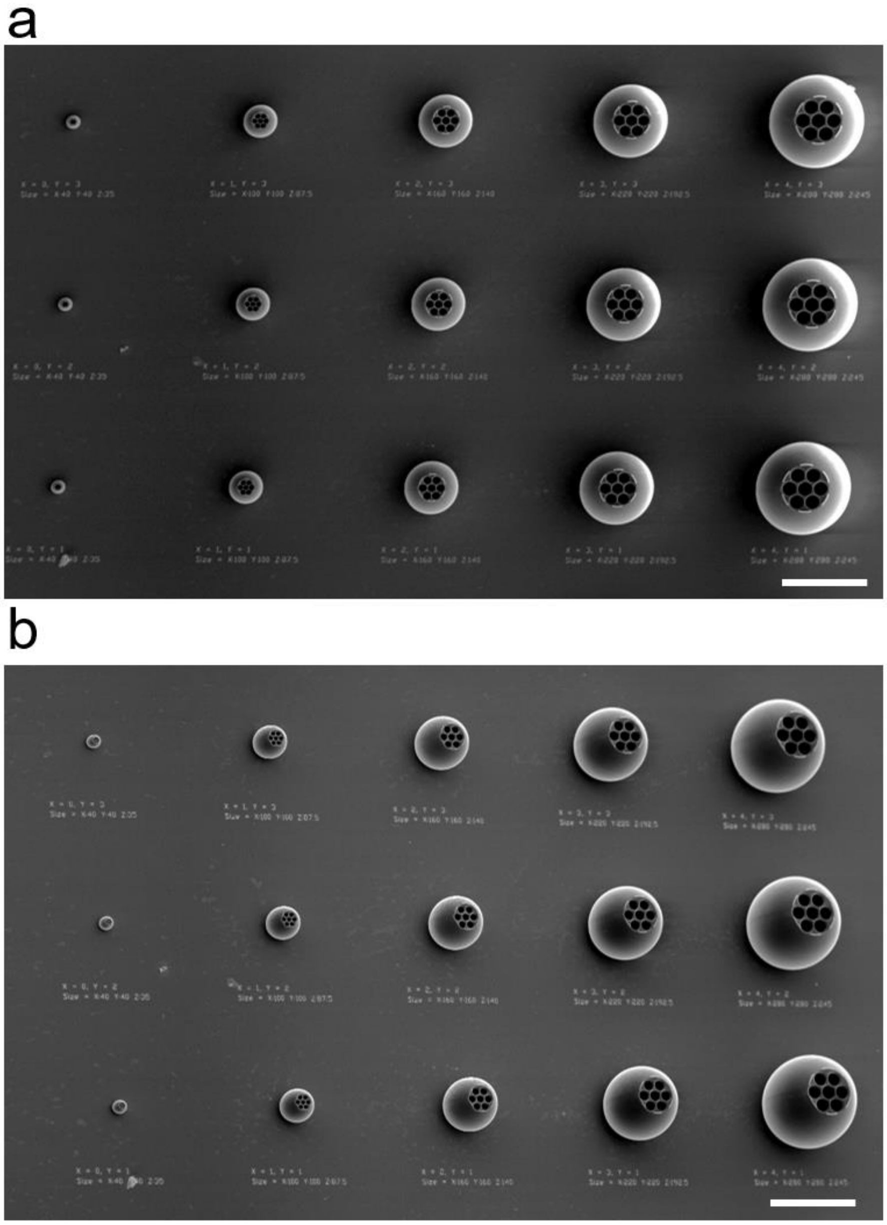

Figure S4. SEM images of the 2-PIMs with different sizes from top view a) and tilted view b). From left to right: body diameter = 40, 100, 160, 220, 280 μm. Scale bars = 250 μm.

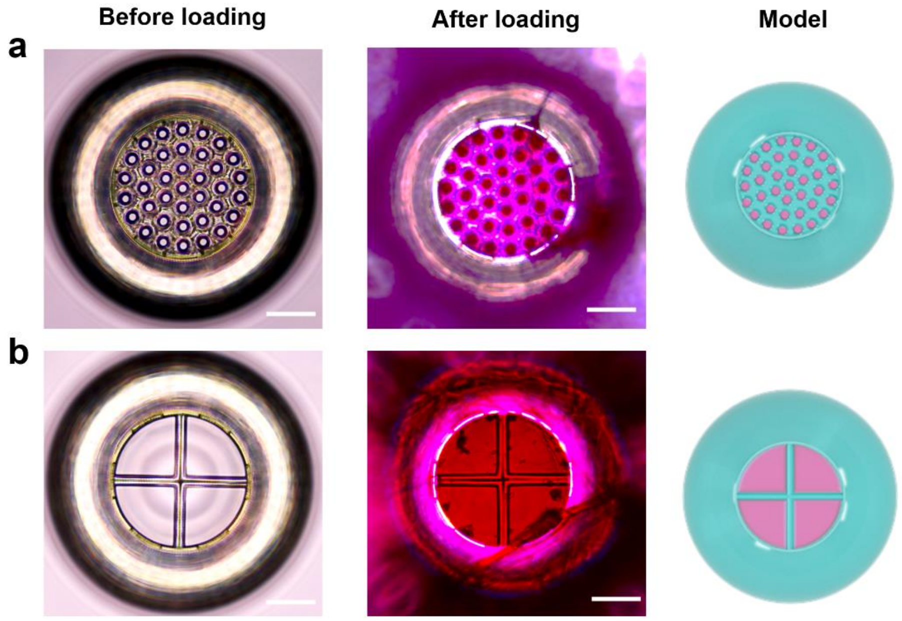

Figure S5. Brightfield microscopy images of 1-PIM (a) and 3-PIM (b). From left to right: brightfield microscopy images of PIMs before loading, brightfield microscopy images of PIMs after loading, and models of loaded PIMs. Scale bars = 50 μm.

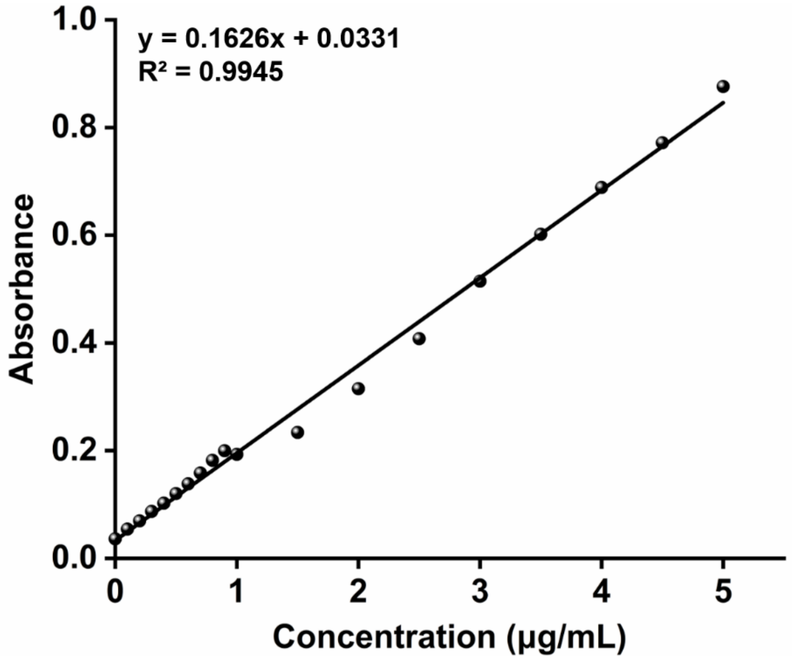

Figure S6. Standard curve of RhB for quantitative analysis. Data shown as mean of n = 8 technical replicates.

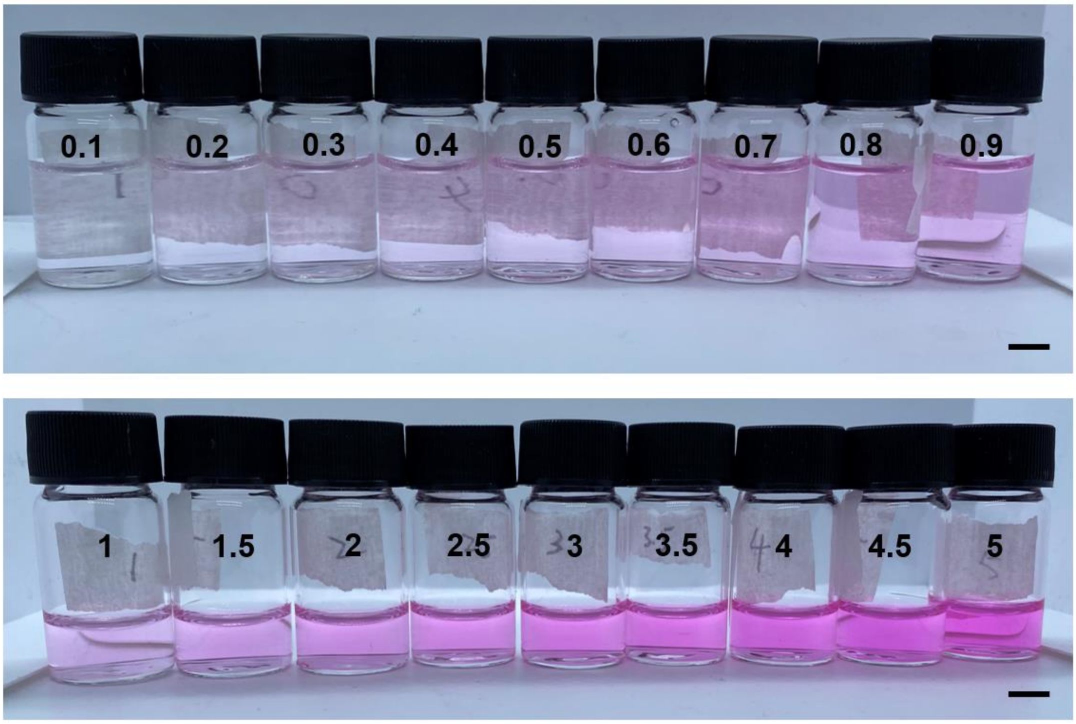

Figure S7. Gradient dilution of RhB solutions for obtaining the standard curve. Scale bars = 5 mm.

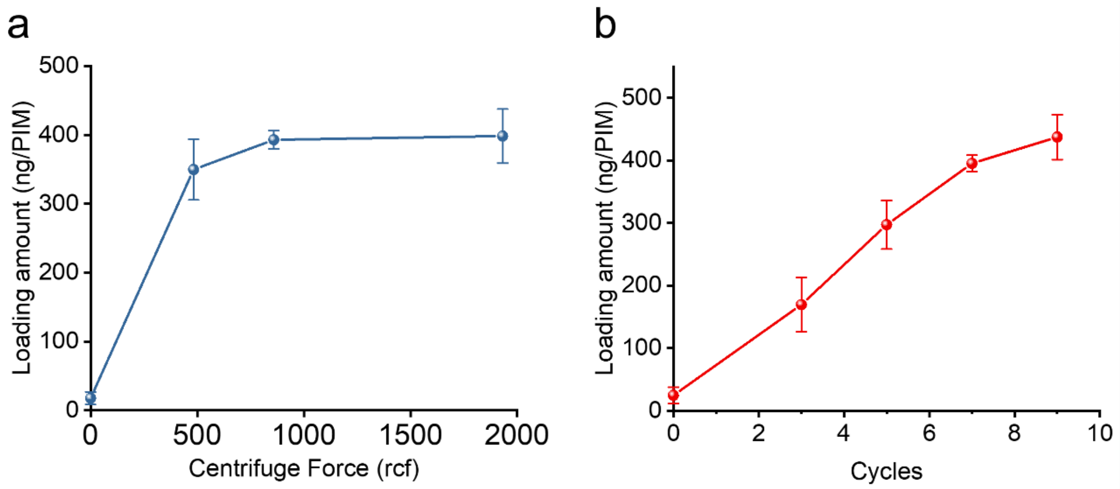

Figure S8. a) RhB loading quantities for 2-PIM at different centrifuge forces. b) RhB loading quantities for 2-PIM at different centrifuge cycles. All values are expressed as mean ± SD, *n* = 3.

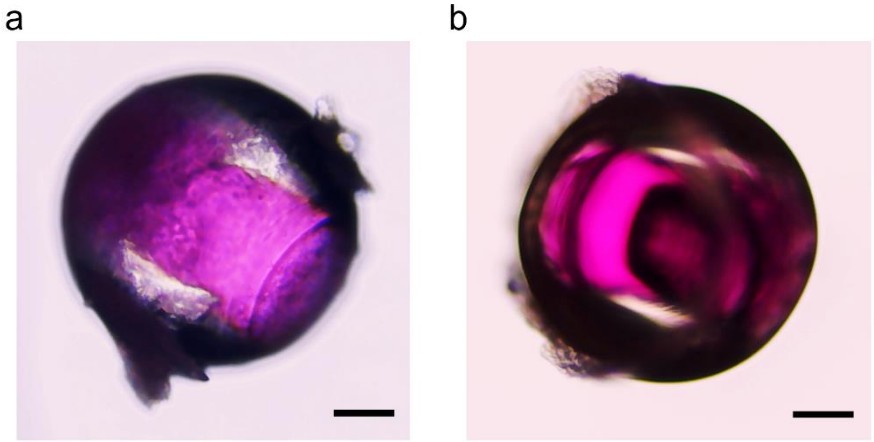

Figure S9. Brightfield microscopy images of the 2-PIM loaded with (a) RhB/PEG 400 and (b) RhB/DMSO. Scale bars = 50 μm.

Table S1. Evaluation of different types of sealing materials. Good adhesion properties indicated the sealing materials would not detach from the PIMs. Good water proof properties indicated the sealing materials would be able to prevent moisture ingress. Good film-forming properties indicated the sealing materials would be able to form a uniform film by spin-coating.

Materials	Phase-change temperature	Adhesion	Water proof	Film-forming
Gelatin	40 ºC	Good	Bad	Bad
Carrageenan	60 ºC	Good	Bad	Bad
1-tetradecanol	39 ºC	Bad	Good	Bad
Polycaprolactone (High Mw)	70 ºC	Good	Good	Good
Polycaprolactone diol	53 ºC	Good	Good	Good

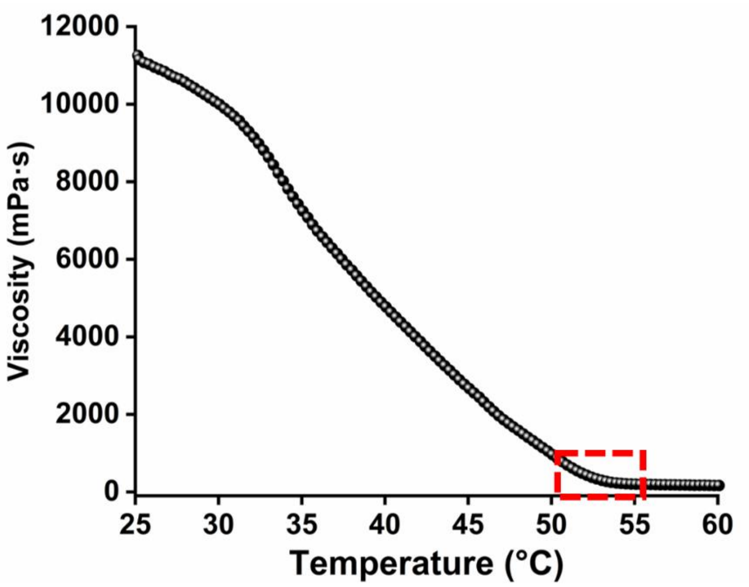

Figure S10. Viscosity-temperature curve for PCL diol. Melting was observed around 50-55 °C (red).

Table S2. Evaluation of different types of NIR photothermal agents. Good photothermal abilities indicated the photothermal agents could melt PCL diol under NIR irradiation. Good adhesion/miscibility indicated the photothermal agents would not detach from PCL diol. Good uniformity indicated the photothermal agents dispersed uniformly in PCL diol.

Strategies	Photothermal abilities	Adhesion/ miscibility	Uniformity
Gold sputtering[3]	Good	Bad	Good
Blending with polypyrrole nanoparticles (PPy NPs)	Good	Bad	Bad
Spray coating of PPy NPs	Bad	Bad	Bad
PPy vapour polymerization[4]	Bad	Good	Good
Pd/Fe_3O_4 coating[5]	Bad	Good	Bad
Spray coating of Fe_3O_4 NPs[6]	Bad	Good	Good
Blending with IR-780	Good	Good	Good

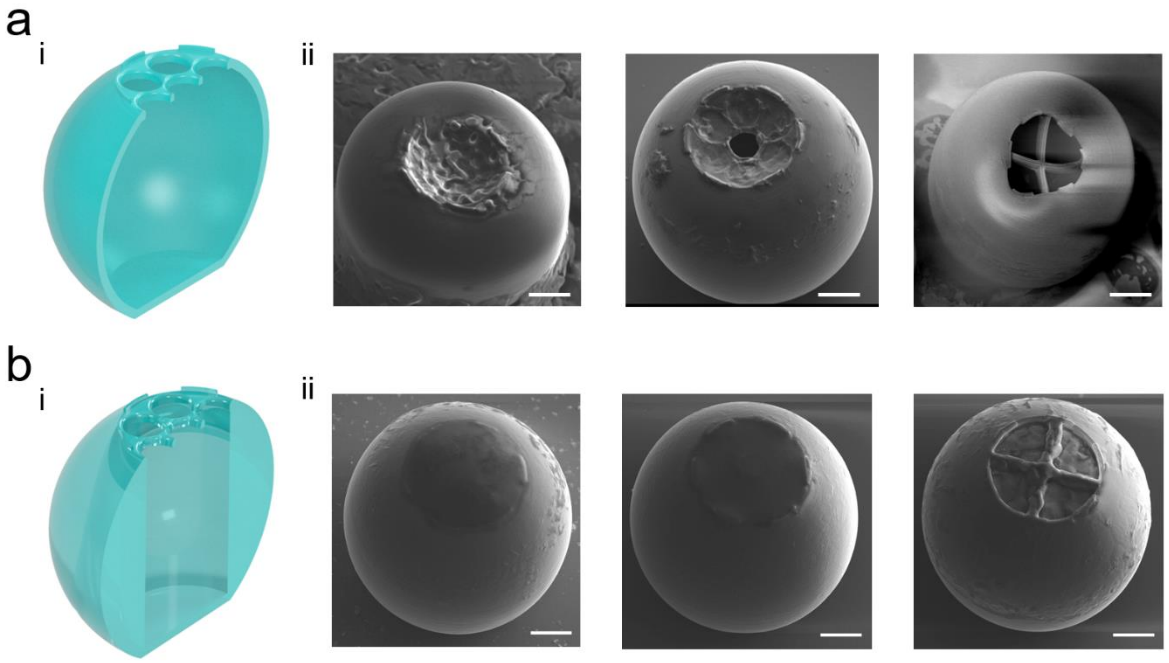

Figure S11. a) i: Schematic diagram of the 2-PIM with thin-walled shell cavity. ii: from *left* to *right*, the SEM images for sealed 1-PIM, 2-PIM, and 3-PIM. Scale bars = 50 μm. b) i: Schematic diagram of the PIM with a cylindrical internal cavity. ii: from *left* to *right*, the SEM images for sealed 1-PIM, 2-PIM, and 3-PIM. Scale bars = 50 μm.

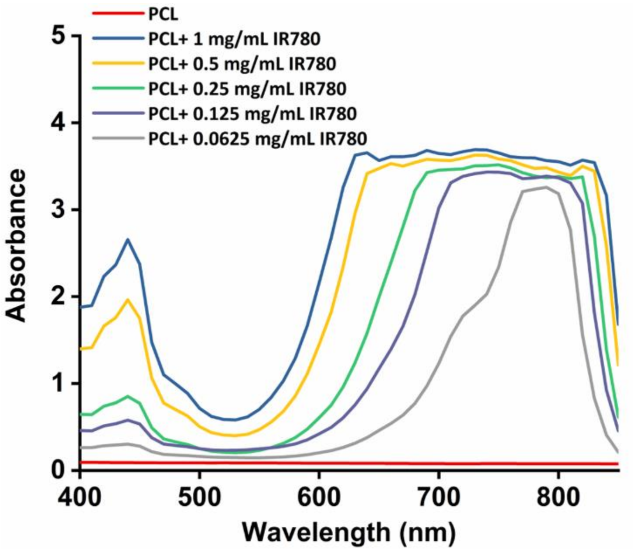

Figure S12. UV–vis–NIR absorption spectra of PCL and PCL-IR780 with different blending concentrations.

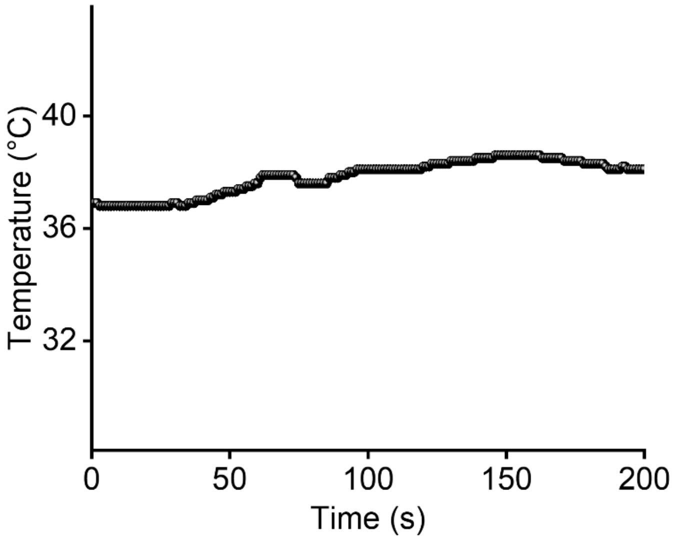

Figure S13. Photothermal heating curves of the surrounding environment under NIR laser irradiation on PIMs (808 nm, 0.6 W cm^{-2}) over 200 seconds.

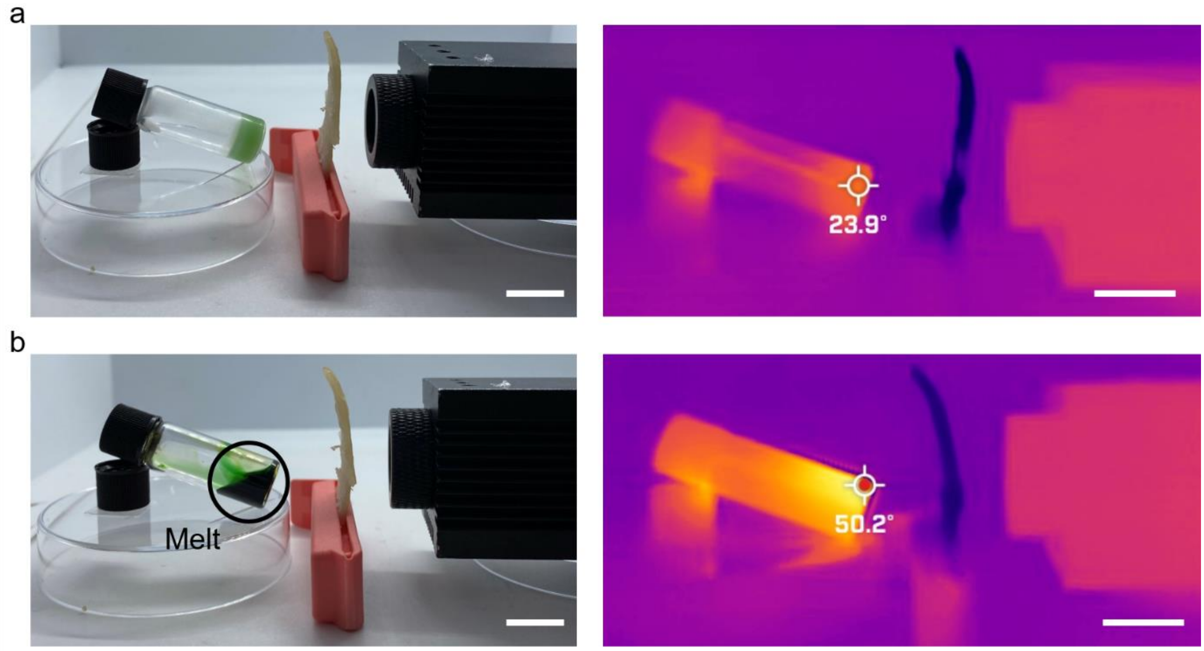

Figure S14. The photos (*left*) and infrared thermal images (*right*) of PCL with different concentrations of IR780: 0.1 mg/mL for panel a) and 10 mg/mL for panel b) after NIR irradiation (808 nm, 0.6 W cm^{-2}). Scale bars = 1 cm.

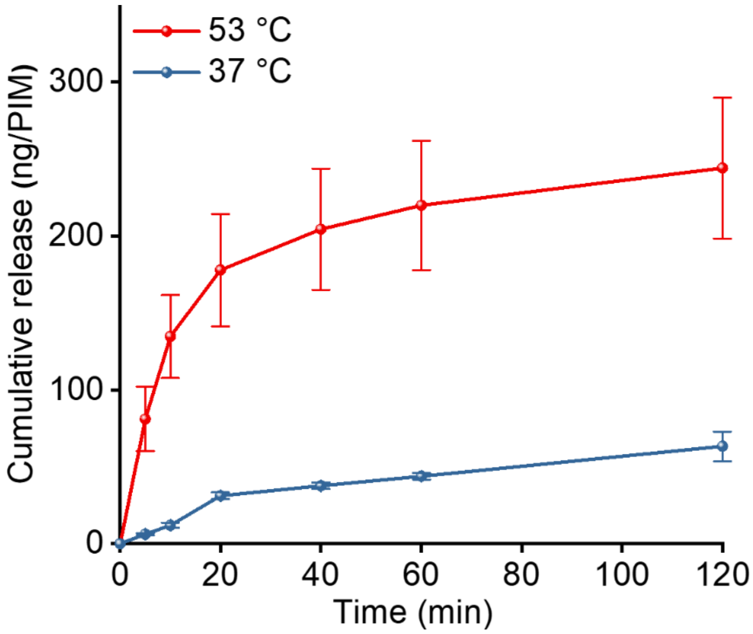

Figure S15. Cumulative RhB release profile from 2-PIMs at 37 °C and 53 °C. All values are expressed as mean ± SD, n = 3.

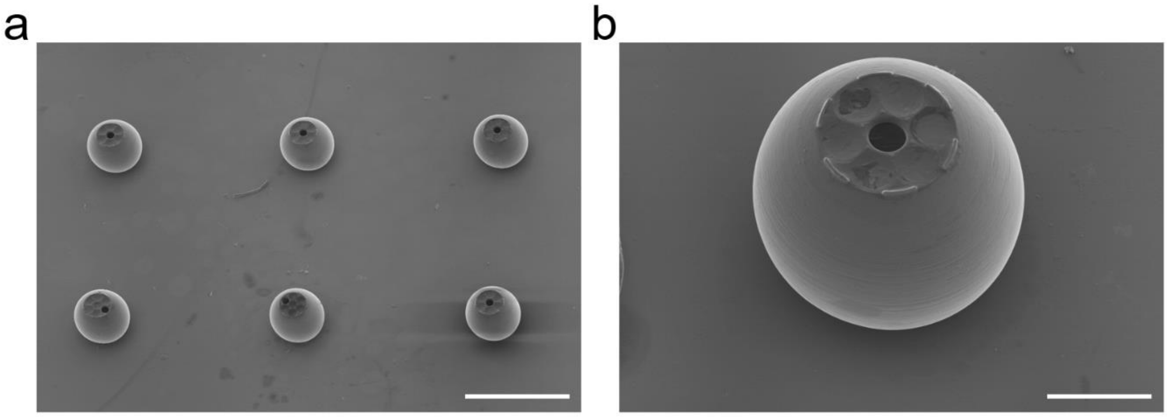

Figure S16. SEM images of the a) 2-PIM array and b) a single 2-PIM after one NIR on/off cycle. Scale bars from left to right, 500 μm and 100 μm.

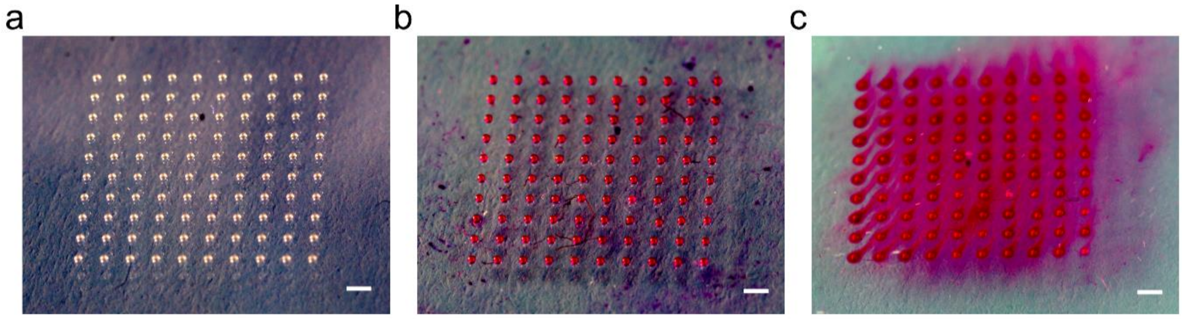

Figure S17. Photos of the 10×10 array of PIMs after printing (a), after loading with RhB solution (b), and after triggering the release of the loaded RhB. Scale bars = 1 mm.

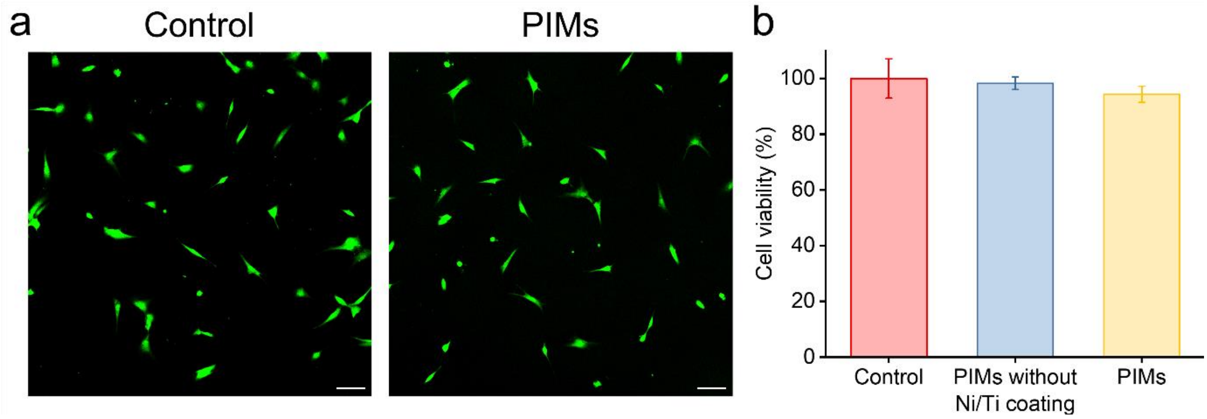

Figure S18. a) LIVE/DEAD^{TM} staining images of the cells: (*left*) no treatment and (*right*) treated with PIMs. Scale bar = 50 µm. b) Cell viability after 24 h of incubation PIMs with or without Ni/Ti coatings using the AlamarBlue® assay. Cells cultivated on tissue culture plate were set as control (*n* = 12). The cell viability values for PIMs are expressed as mean ± SD, *n* = 3.

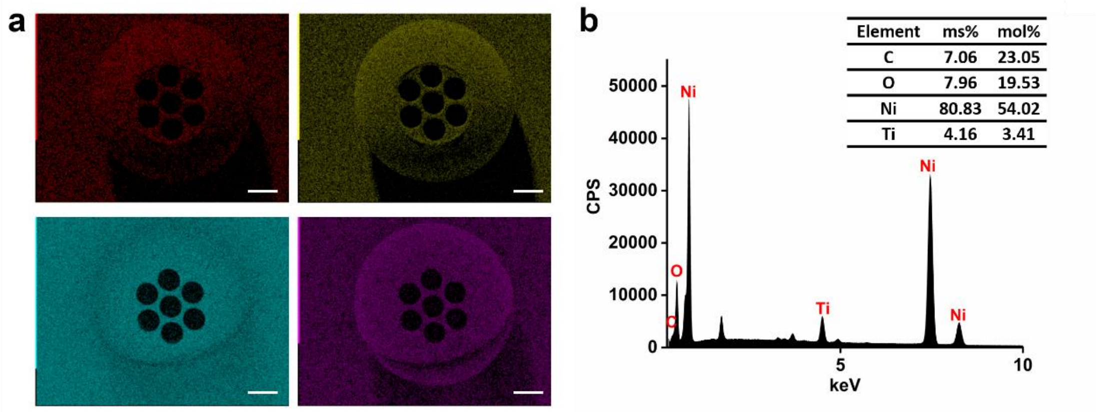

Figure S19. a) EDX mapping of the PIMs after Ni/Ti physical vapour deposition from the top view (tilted view shown in Figure 4a). Each element shown in a different colour. Red: carbon, yellow: oxygen, cyan: nickel, purple: titanium. Scale bars = 50 μm. b) Energy dispersive spectra (EDS) of PIMs after Ni/Ti deposition from the top view. Mass fractions (wt. %) and atom fractions (at. %) of C, O, Ni and Ti elements of the PIMs were shown in the inserted table.

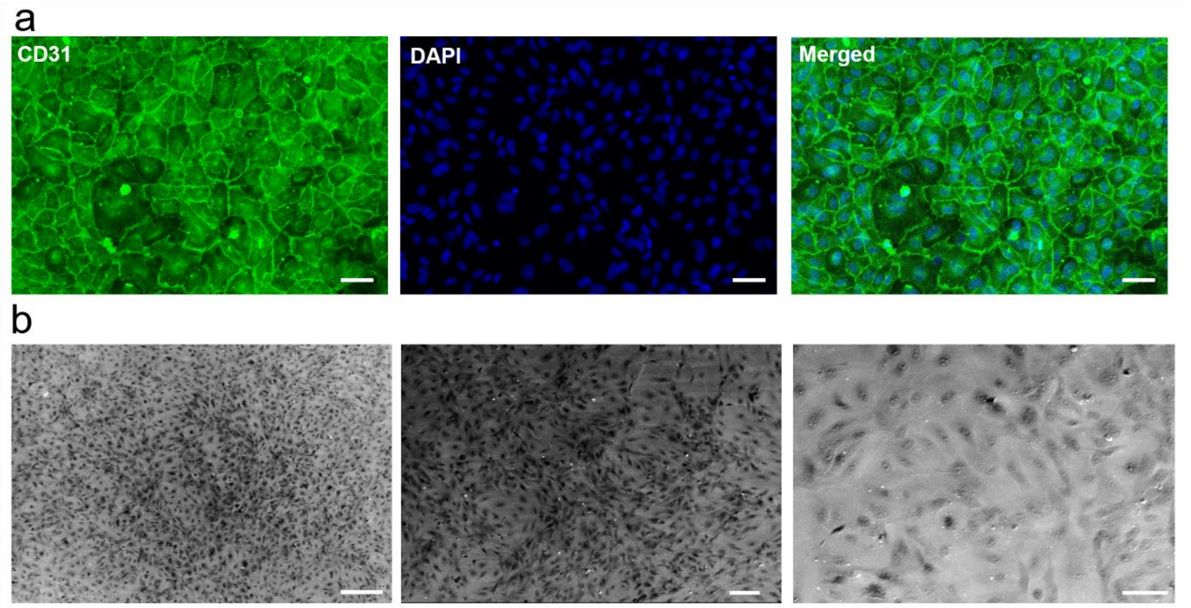

Figure S20. a) From *left* to *right*: CD31 staining, DAPI staining, and merged confocal images of the cell-adhered surfaces. Scale bars = 50 μm. b) SEM images of the cell-adhered surfaces. Scale bar from *left* to *right*: 200, 100, and 50 μm.

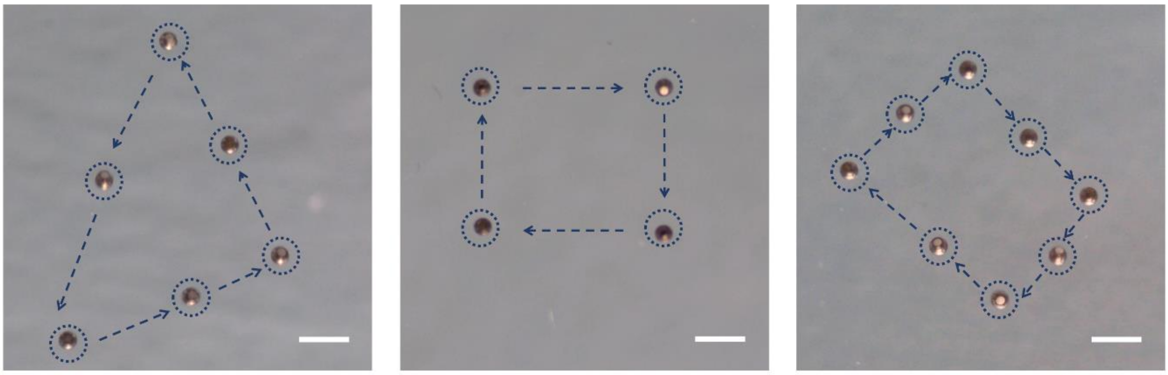

Figure S21. Time-lapse trajectory snapshots of controlled locomotion of PIMs along desired paths (triangle, rectangle, and rhombus) under rotating magnetic fields (2 mT, 5 Hz). Scale bars = 500 μm.

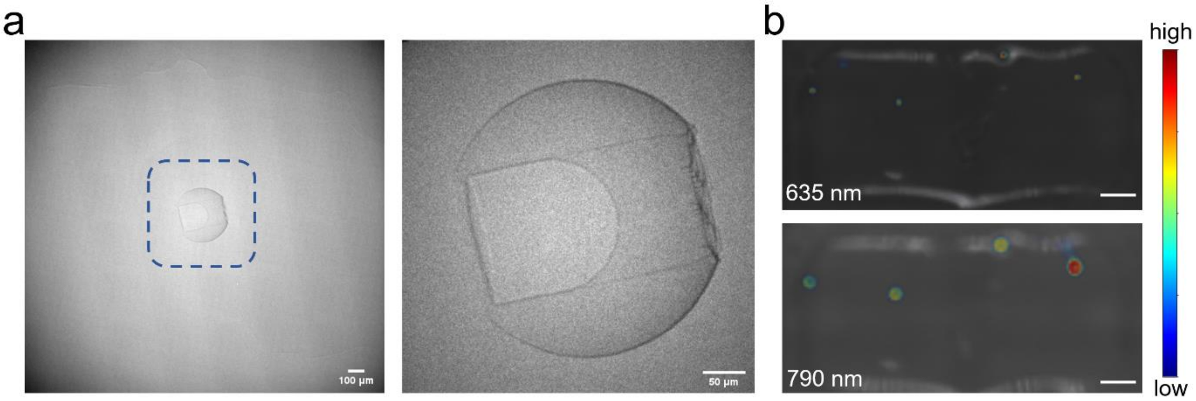

Figure S22. a) X-ray imaging of the PIM inside the agarose hydrogel (2 wt.%). Scale bars from left to right: 100 μm and 50 μm. b) Fluorescence molecular tomography (FMT) of the PIMs inside the agarose hydrogel (2 wt.%) under two wavelengths (*top*: 635 nm, *bottom*: 790 nm). The color scale bar represents fluorescence signal intensity. Scale bars = 10 mm.

References

[1] a) M. Dong, X. Wang, X. Z. Chen, F. Mushtaq, S. Deng, C. Zhu, H. Torlakcik, A. Terzopoulou, X. H. Qin, X. Xiao, *Advanced Functional Materials* **2020**, 30, 1910323; b) A. Marino, S. Arai, Y. Hou, E. Sinibaldi, M. Pellegrino, Y.-T. Chang, B. Mazzolai, V. Mattoli, M. Suzuki, G. Ciofani, *ACS Nano* **2015**, 9, 7678.
[2] M. Minekus, M. Alminger, P. Alvito, S. Ballance, T. Bohn, C. Bourlieu, F. Carrière, R. Boutrou, M. Corredig, D. Dupont, *Food & Function* **2014**, 5, 1113.
[3] Z. Wu, L. Li, Y. Yang, P. Hu, Y. Li, S.-Y. Yang, L. V. Wang, W. Gao, *Science Robotics* **2019**, 4, eaax0613.
[4] J. E. Yim, S. H. Lee, S. Jeong, K. A. Zhang, J. Byun, *Journal of Materials Chemistry A* **2021**, 9, 5007.
[5] a) R. Hensleigh, H. Cui, Z. Xu, J. Massman, D. Yao, J. Berrigan, X. Zheng, *Nature Electronics* **2020**, 3, 216; b) T. Zandrini, S. Taniguchi, S. Maruo, *Micromachines* **2017**, 8, 35.
[6] J. Tu, W. Gao, *Science Robotics* **2020**, 5, eabf1390.
